# Persistence and decay of neutralizing antibody responses elicited by SARS-CoV-2 infection and hybrid immunity in a Canadian cohort

**DOI:** 10.1128/spectrum.01333-24

**Published:** 2025-02-19

**Authors:** Amy Nouanesengsy, Anthony Semesi, Kim Quach, Danton Ivanochko, Walter Byrne, Matthew Hwang, Maria-Rosa La Neve, Matilde Leon-Ponte, Alice Litosh, Nicole Wisener, Khosrow Adeli, Aaron Campigotto, Eyal Grunebaum, Allison McGeer, Theo J. Moraes, Lusia Sepiashvili, Julia Upton, Jean-Philippe Julien, Upton Allen

**Affiliations:** 1 Program in Molecular Medicine, The Hospital for Sick Children, Research Institute, Toronto, Ontario, Canada; 2 Department of Biochemistry, University of Toronto, Toronto, Ontario, Canada; 3 Department of Paediatric Laboratory Medicine, The Hospital for Sick Children 7979 , Toronto, Ontario, Canada; 4 Division of Infectious Diseases, Department of Paediatrics, The Hospital for Sick Children, Toronto, Ontario, Canada; 5 Division of Allergy and Immunology, Department of Paediatrics, The Hospital for Sick Children, Toronto, Ontario, Canada; 6 Department of Laboratory Medicine and Pathobiology, University of Toronto 7938 , Toronto, Ontario, Canada; 7 Developmental and Stem Cell Biology Program, The Hospital for Sick Children 7979 , Toronto, Ontario, Canada; 8 Child Health Evaluative Sciences, The Hospital for Sick Children, Research Institute, Toronto, Ontario, Canada; 9 Lunenfeld-Tunenbaum Research Institute at Mount Sinai Hospital, Sinai Health 518775 , Toronto, Ontario, Canada; 10 Translational Medicine Program, The Hospital for Sick Children 7979 , Toronto, Ontario, Canada; 11 Department of Pediatrics, Temerty Faculty of Medicine, University of Toronto, Toronto, Ontario, Canada; 12 Department of Immunology, University of Toronto 7938 , Toronto, Ontario, Canada; Oklahoma State University College of Veterinary Medicine, Stillwater, Oklahoma, USA

**Keywords:** SARS-CoV-2, neutralizing antibodies, hybrid immunity

## Abstract

A major challenge with severe acute respiratory syndrome coronavirus 2 (SARS-CoV-2), the causative agent of coronavirus disease 2019 (COVID-19), has been assessing the intensity, dynamics, and determinants of the antibody responses after infection and/or vaccination. Therefore, we aimed to characterize the longitudinal dynamics of the antibody responses among naturally infected individuals and individuals who achieved hybrid immunity in a large Canadian cohort. We demonstrate that anti-Spike IgGs and neutralizing antibody dynamics vary greatly among individuals with COVID-19, in peak antibody levels, rate of waning, and longevity of the antibody response. Additionally, we found an association between robust antibody responses and individuals with severe COVID-19 clinical symptoms during the first-month post-symptom onset. For individuals who achieved hybrid immunity, a robust increase in anti-S1 IgGs and neutralizing antibodies followed the first vaccination dose; however, there was a minimal increase in the anti-S1 IgGs and neutralizing antibody titers after administration of the second dose of the vaccine. Furthermore, neutralizing antibodies elicited by the wild-type virus alone were largely ineffective against emerging variants of concern in our natural infection-only cohort, in contrast to a much broader and more robust neutralization profile observed in individuals who achieved hybrid immunity. Our findings emphasize the need for global SARS-CoV-2 vaccination efforts to further sustain protective immune responses required to minimize viral spread and disease severity in the population. As SARS-CoV-2 variants continue to emerge, understanding the interplay between previous infections, vaccine durability, and virus evolution will be critical for guiding ongoing vaccination strategies.

**IMPORTANCE:**

A major challenge with severe acute respiratory syndrome coronavirus 2 (SARS-CoV-2), the causative agent of coronavirus disease 2019 (COVID-19), has been assessing the intensity, dynamics, and determinants of the antibody response after infection and/or vaccination. Our paper addresses this in a large Canadian cohort with antibody responses that were generated by natural infection as well as vaccine in some persons studied.

## INTRODUCTION

Coronavirus disease 2019 (COVID-19), caused by the severe acute respiratory syndrome coronavirus 2 (SARS-CoV-2), has resulted in more than 6.9 million deaths worldwide as of December 2023 (1). Several COVID-19 vaccines have received WHO EUA status, and more than 3.4 billion vaccine doses have been administered worldwide (1). Since the emergence of SARS-CoV-2 and the development and approval of these vaccines, a key priority has been determining the quality and durability of the immune response. Several studies have suggested that the quality and duration of the adaptive immune response differ depending on whether they were induced by natural infection, vaccine, or a combination of both modalities (hybrid immunity). In the case of the latter, the determination of immunity may occur after individuals develop natural immunity from infection and then are subsequently vaccinated with or without further episodes of infection, or are vaccinated before experiencing natural infection (2, 3). The antibody (Ab) response, specifically SARS-CoV-2-binding IgG concentrations in the serum and antibody functionality, particularly their ability to neutralize the SARS-CoV-2 virus, serve as a correlate of protection against infection. The thresholds for these immunological protection mechanisms have been defined for certain SARS-CoV-2 variants of concern (VOCs) (4).

SARS-CoV-2 infection is mediated by the viral spike protein (S), which is composed of the S1 and S2 subunits (5). The receptor-binding domain (RBD) within the S1 subunit allows for virus attachment to the host cell receptor, human angiotensin-converting enzyme 2 (ACE2), to mediate infection (6). Antibodies generated from the humoral immune response can elicit a multitude of effector functions, which harness immune cells to eliminate the virus (7). Importantly, antibodies can also bind to the virus and directly prevent it from infecting host cells, thereby neutralizing the virus and inhibiting further viral replication (6). Analysis of the antibody responses across multiple cohorts of patients who have recovered from COVID-19 shows that natural infection can elicit neutralizing antibodies in most cases, but accumulating evidence indicates that the magnitude of the response varies greatly across individuals (8, 9). Several studies have suggested that the differences in antibody responses may be due to the broad spectrum of clinical manifestations experienced by patients (10–12).

The heterogeneity in the antibody response among the population has complicated the ability to predict whether individuals have achieved protective immunity. Adding to this complexity, the SARS-CoV-2 virus exhibits rapid mutations in its spike protein. Several VOCs have emerged, making the virus more transmissible and less susceptible to neutralizing antibodies found in convalescent or post-vaccination sera. This has led to an increase in both reinfections and breakthrough infections (13–15).

In this study, we first aimed to characterize the antibody kinetics and neutralizing antibody capacity from 321 patient sera in a Canadian cohort where they were characterized as naturally infected at the very beginning of the pandemic. These patient samples were collected at different time points post-symptom onset (PSO) before the introduction and availability of the SARS-CoV-2 vaccines. Second, in our natural infection-only cohort, we examined how disease severity affected the antibody responses, as well as how the responses changed during the different phases of the infection. Third, we compared the neutralizing capacity of antibodies among persons with natural infection only compared to those who were naturally infected and subsequently vaccinated approximately a year later (hybrid cohort). Lastly, we assessed the neutralizing capacity of antibodies from individuals infected by the wild-type strain of the virus against the different VOCs before and after vaccination to assess the durability and resilience of the antibody response.

## RESULTS

### Characteristics of the study cohorts

Samples were collected at The Hospital for Sick Children, Mount Sinai Hospital, and North York General Hospital in Toronto, Ontario, Canada. The samples were divided into three cohorts: (i) symptomatic patients who tested positive for SARS-CoV-2 by PCR or by a serology assay (cohort A; *n* = 178, 321 samples), (ii) symptomatic patients who tested positive for SARS-CoV-2 by PCR or by a serology assay and had been subsequently vaccinated (cohort B; *n* = 46, 48 samples), and (iii) healthy naïve controls (cohort C; *n* = 21, 21 samples) (Table 1).

**TABLE 1:** Characteristics of study cohort

	Cohort A: natural infection *n* = 321 (82%)	Cohort B: hybrid immunity (natural infection + COVID-19 vaccinated) *n* = 48 (12%)	Cohort C: naïve control *n* = 21 (6%)
Age, years, median (range)	42.0 (0.25–100.0)	43.0 (19.0–76.0)	16.0 (0.25–70.0)
<2, *n* (%)	10 (3)	^*e*^-	2 (9)
2–5, *n* (%)	4 (1)	-	1 (5)
6–11, *n* (%)	26 (8)	-	6 (29)
12–17, *n* (%)	22 (7)	-	2 (9)
≥18, *n* (%)	259 (81)	48 (100)	10 (48)
Females, *n* (%)	157 (49)	30 (62)	9 (43)
COVID-19 diagnostic			
PCR positive, *n* (%)	252 (79)	45 (94)	0 (0)
Seropositive, *n* (%)	223 (69)	48 (100)	5 (24)
Clinical severity			
Mild^*a*^	211 (66)	40 (83)	-
Hospitalized (mild)^*b*^	60 (19)	7 (15)	-
Hospitalized (severe)^*c*^	39 (12)	1 (2)	-
Death^*d*^	11 (3)	0 (0)	-
Asymptomatic	13 (4)	0 (0)	21 (100)
Symptomatic	308 (96)	48 (100)	0 (0)
Days post-symptom onset, days, median (range)	55 (2–383)	24 (4–91)	-
Early acute (0–14 days), *n* (%)	74 (23)	1 (2)	-
Late acute (15–27 days), *n* (%)	36 (11)	0 (0)	-
Early convalescent (4–12 weeks), *n* (%)	69 (22)	14 (29)	-
Late convalescent (>12 weeks), *n* (%)	142 (44)	33 (69)	-
Vaccination			
AstraZeneca (Vaxzevria) (viral vector), *n* (%)	-	2 (4)	-
Moderna (SpikeVax) (mRNA), *n* (%)	-	4 (8)	-
Pfizer/BioNTech (Comirnaty) (mRNA)	-	42 (88)	-
Number of vaccine doses			
One dose, *n* (%)	-	40 (83)	-
Two doses, *n* (%)	-	8 (17)	-
Days post-vaccination, days, median (range)	-	23.5 (4–91)	-

^*a*^ A clinical severity score of 1 and 2 defined by the World Health Organization (WHO) (16).

^*b*^ A clinical severity score of 3 and 4 defined by the WHO.

^*c*^ A clinical severity score of 5, 6, and 7 defined by the WHO.

^*d*^ A clinical severity score of 8 defined by the WHO.

^*e*^ “-”, no samples.

In our natural infection cohort A (naturally infected SARS-CoV-2 positive cases), the majority of cases had mild symptoms (*n* = 211 samples, 66%) with a clinical severity score of 1 or 2. The clinical severity score for each patient was assessed and determined according to the WHO criteria (16). Nineteen percent (*n* = 60 samples) of the cohort was hospitalized with mild disease and had a clinical severity score of 3 or 4; 12% (*n* = 39) of the cohort was hospitalized with severe disease requiring an intensive care unit (ICU) level of care and had a clinical severity score of 5, 6, or 7; and 3% (*n* = 11 samples) of the cohort died and had a clinical severity score of 8. In cohort A – natural infection, 49% were female (median age of 42 years) with an age range of 3 months to 100 years (Table 1).

Blood and/or serum samples were collected at multiple time points for some patients, and in cohort A, 51% (*n* = 113 individuals) of this cohort had >2 visits. Within cohort A, 23% of the samples were collected during the early acute phase of the infection, which was 0–14 days after the onset of symptoms (*n* = 74); 11% were from the late acute phase between 15 and 27 days (*n* = 36); 22% of the samples were from the early convalescent phase between 4 and 12 weeks (*n* = 69); and 44% were collected during the late acute phase, which were samples collected later than 12 weeks PSO (*n* = 142) (Table 1). The median PSO was 55 days with a range of 2–383 days in cohort A.

In the hybrid immunity cohort B, which included 48 individuals who were naturally infected and subsequently vaccinated with at least one dose of the SARS-CoV-2 vaccines, 62% were female (*n* = 30), with a median age of 43 years, and the overall cohort ranging from 19 to 76 years old (Table 1). Samples were confirmed positive for SARS-CoV-2 via PCR (83%, *n* = 45) and/or a serology assay (100%, *n* = 48). Individuals in cohort B were vaccinated with either AstraZeneca’s Vaxzervria vaccine (4%, *n* = 2), Moderna’s SpikeVax vaccine (8%, *n* = 4), or Pfizer/BioNTech’s Comirnaty vaccine (88%, *n* = 42). Most samples were collected after one dose of the vaccine (83%, *n* = 48), and 17% were collected after two doses of the vaccine (*n* = 8). Samples were collected 4 to 91 days post-vaccination with a median of 24 days in cohort B (Table 1).

Cohort C, which was the naïve control sample, was 43% female (*n* = 9) with a median age of 16 years and an age range of 3 months to 70 years (Table 1).

### Quantity and quality of SARS-CoV-2 antibody response elicited by natural infection

**Fig 1:**
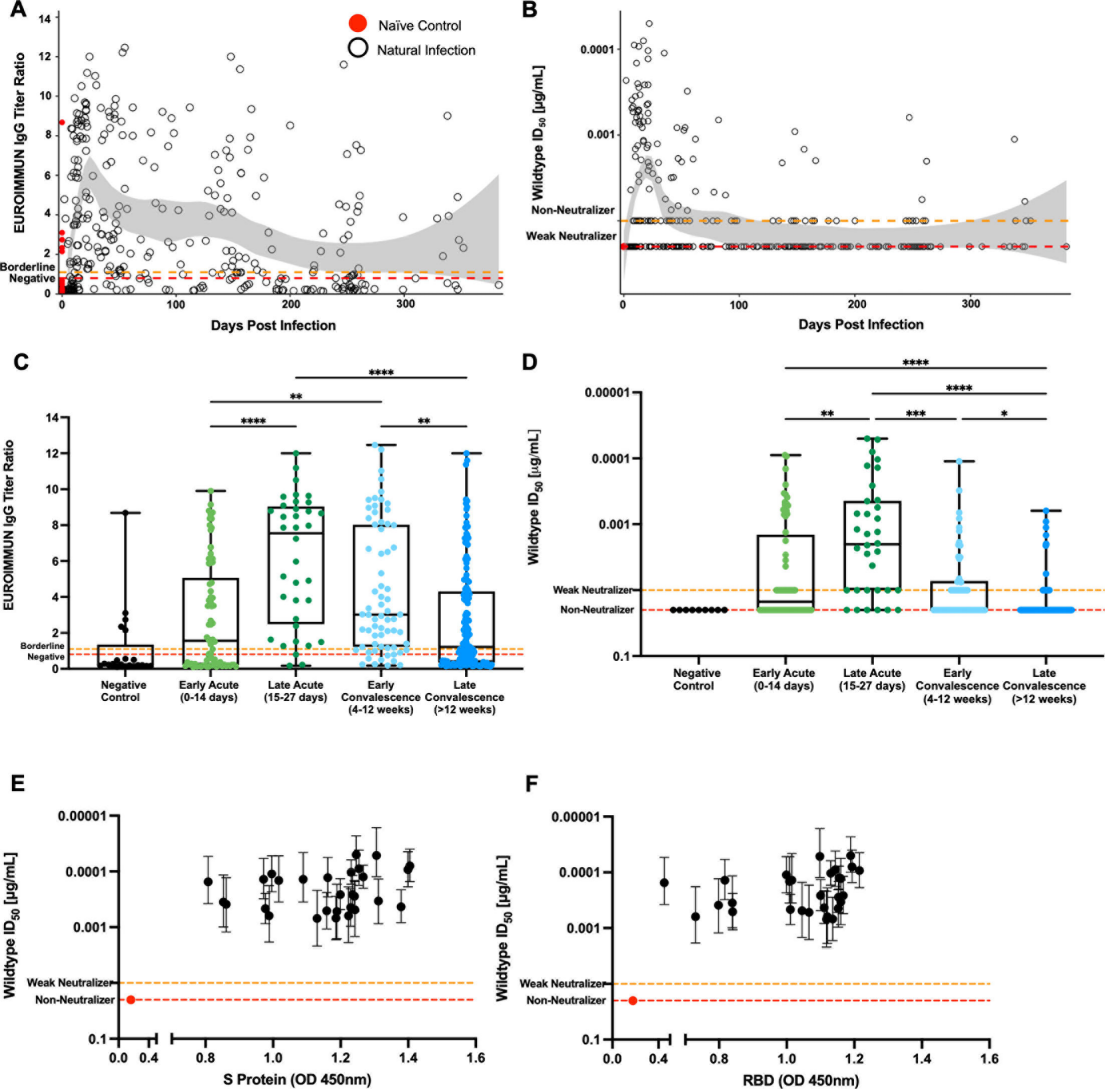
Longitudinal analysis of the antibody responses against the SARS-CoV-2 wild-type (D614G) spike protein from naturally infected individuals. (**A**) 321 longitudinal serum patient samples were collected from 178 patients and 21 control samples, and were tested by the commercial EUROIMMUN SARS-CoV-2 S1 ELISA at a dilution of 1:100 for the presence of SARS-CoV-2 spike-specific IgG antibodies. Each black dot represents a unique measurement of a SARS-CoV-2 patient sample. Each red dot represents a unique measurement of a control, SARS-CoV-2 naïve sample at day 0. The red dotted lines at a ratio of 0.8 signifying those points below the line are negative for anti-Spike IgGs. The orange dotted lines at a ratio of 1.1 signifying the points between the orange and red dotted lines are borderline for anti-Spike IgGs. A LOESS smooth parametric function with a span of 0.5 applied with a 95% confidence interval is shaded in light gray. Positivity for some of the SARS-CoV-2 naïve samples suggests prior infection, cross-reactivity, and/or higher-than-expected assay background. (**B**) 321 patient samples from 178 SARS-CoV-2 positive patients and 22 control individuals were tested by pseudoviral neutralization at a starting dilution of 1:50 of heat-inactivated sera and 1:4 serial dilutions to obtain an ID_50_ value, represented by a black dot that is a unique measurement of a SARS-CoV-2 patient. Each red dot at day 0 represents a unique measurement of a control sample. The orange dotted lines at 0.01 µg/mL represent weak neutralizing samples that can neutralize the WT PsV at a dilution of 1:50, but we are unable to fit a proper sigmoidal curve to the data set; the red dotted lines at an ID_50_ value of 0.02 µg/mL represent non-neutralizing samples, which are samples that do not meet any of the criteria needed to assign an ID_50_ value to the sample. A LOESS smooth parametric function with a span of 0.5 applied with a 95% confidence interval is shaded in light gray. (**C**) Comparison of anti-Spike IgGs in serum in cohorts of pre-COVID samples (*n* = 21), early acute COVID infection (*n* = 74), late acute COVID infection (*n* = 36), early convalescent COVID infection (*n* = 68), and late convalescent COVID infection (*n* = 141). Comparisons between groups were carried out by the ANOVA-Kruskal-Wallis test (*P < 0.05, **P < 0.01, ***P < 0.001, ****P < 0.0001). (**D**) Comparison of neutralizing antibodies against the wild-type PsV in serum in cohorts of pre-COVID samples (*n* = 9), early acute COVID infection (*n* = 68), late acute COVID infection (*n* = 34), early convalescent COVID infection (*n* = 54), and late convalescent COVID infection (*n* = 107). Comparisons between groups were performed by the ANOVA-Kruskal-Wallis test (*P < 0.05, **P < 0.01, ***P < 0.001, ****P < 0.0001). Twenty-nine of the top neutralizing SARS-CoV-2 serum samples from the cohort and one control sample were tested by ELISA at a dilution of 1:50 dilution for the presence of SARS-CoV-2 total spike protein-specific (**E**) and RBD-specific (**F**) total antibodies. The bars for each point show the 95% confidence interval.

Serum samples collected from cohort A (natural infection) at different time points were tested to determine the anti-Spike IgG titer ratio using the commercial EUROIMMUN IgG assay. During the early acute phase (0 to 14 days PSO) (*n* = 74), 57% (*n* = 42) of the samples had IgG titer ratios that were higher than baseline, meaning these samples were positive for anti-Spike IgGs in the serum but in varying amounts based on the EUROIMMUN titer ratio (Fig. 1A and C). From days 7 to 14, there was a sharp rise in spike-specific IgGs, and IgG concentrations continued to rise until the end of the acute phase of the infection (*n* = 36) (27 days after the onset of symptoms), where 92% (*n* = 33) of the samples were above baseline. During the acute phase of the infection, a wide range of EUROIMMUN IgG titer ratios was observed for the different samples, and for one sample, anti-Spike IgGs reached an EUROIMMUN ratio of 12 on day 24. The anti-Spike IgG levels were sustained until the end of the early convalescent phase (3 months PSO). During the early convalescent phase (*n* = 69), 88% of the samples (*n* = 61) were above baseline (Fig. 1A and C). The highest level of anti-Spike IgGs was observed in this cohort on day 55 with an EUROIMMUN titer of 12.5. Three months PSO (*n* = 141 samples), anti-Spike IgG titers began to wane (Fig. 1A and C), where only 62% of the samples (*n* = 88) were above baseline. Samples in the late convalescent phase seemed to have lower EUROIMMUN IgG titers with a smaller range among the samples. From the subset of cohort A who had multiple visits, the longitudinal kinetics of the anti-Spike IgG response were similar to the kinetics observed when looking at independent values cross-sectionally (Fig. S1A).

We next looked at the neutralizing capacity of serum from cohort A using a pseudovirus (PsV) neutralization assay against the wild-type SARS-CoV-2 virus. Serum samples from naturally infected patients displayed a broad range of ID_50_ values ranging from >0.02 (non-neutralizing) to 5.016 × 10^−5^ µg/mL against the wild-type PsV. The neutralization status of all samples was divided into one of three groups: non-neutralizers (with an ID_50_ value of >0.02 µg/mL), weak neutralizers (with an ID_50_ value of 0.01 µg/mL), and neutralizers (with ID_50_ values determined by the assay; see Materials and Methods for criteria used for neutralization status classification). A subset of the samples (*n* = 26, 35%) strongly neutralized the wild-type PsV during the early acute phase where ID_50_ values spanned three log units from 0.00436 to 9.27 × 10^−5^ µg/mL, while 17% (*n* = 13) were weak neutralizers (Fig. 1B and D). During the late acute phase (*n* = 36), 61% (*n* = 22) were neutralizers with ID_50_ values ranging from 0.00426 to 5.02 × 10^−5^ µg/mL, and 22% (*n* = 8) were weak neutralizers. The top neutralizer of cohort A was observed during the late acute phase of the infection on day 26 with an ID_50_ value of 5.02 × 10^−5^ µg/mL. During the early convalescent (*n* = 69) and the late convalescent (*n* = 141) phases of the infection, samples above baseline decreased where only 30% (*n* = 21) and 7% (*n* = 10) were neutralizers, respectively, and 20% (*n* = 14) and 16% (*n* = 23) were weak neutralizers, respectively. Looking longitudinally at the 46 individuals who had multiple visits during the study (*n* = 136 samples), 24 individuals developed neutralizing antibodies at some point during the infection, 10 individuals developed a weak neutralizing antibody response, while 12 individuals did not develop a neutralizing antibody response during any of their tested visits (Fig. S1B). Neutralizing antibody kinetics of the longitudinal subset of cohort A were similar to the kinetics observed at the population level, confirmed a progressive decrease, and eventually sustained levels or no presence of neutralizing antibodies during time points sampled after the acute phase and the early convalescent phase of the infection (Fig. 1B and D; Fig. S1B).

For the top neutralizing samples in the natural infection cohort A (*n* = 29), antibodies present in the serum bound to both the entire wild-type spike protein (Fig. 1E) and the RBD (Fig. 1F), showing that antibodies were mounted against the whole spike protein and the RBD of the virus during natural infection. In this cohort, neutralization was positively correlated with the IgG titers with an R^2^ value of 0.52 (Fig. S2A).

### Anti-S1 IgGs and neutralizing antibody responses are associated with the severity of the disease

**Fig 2:**
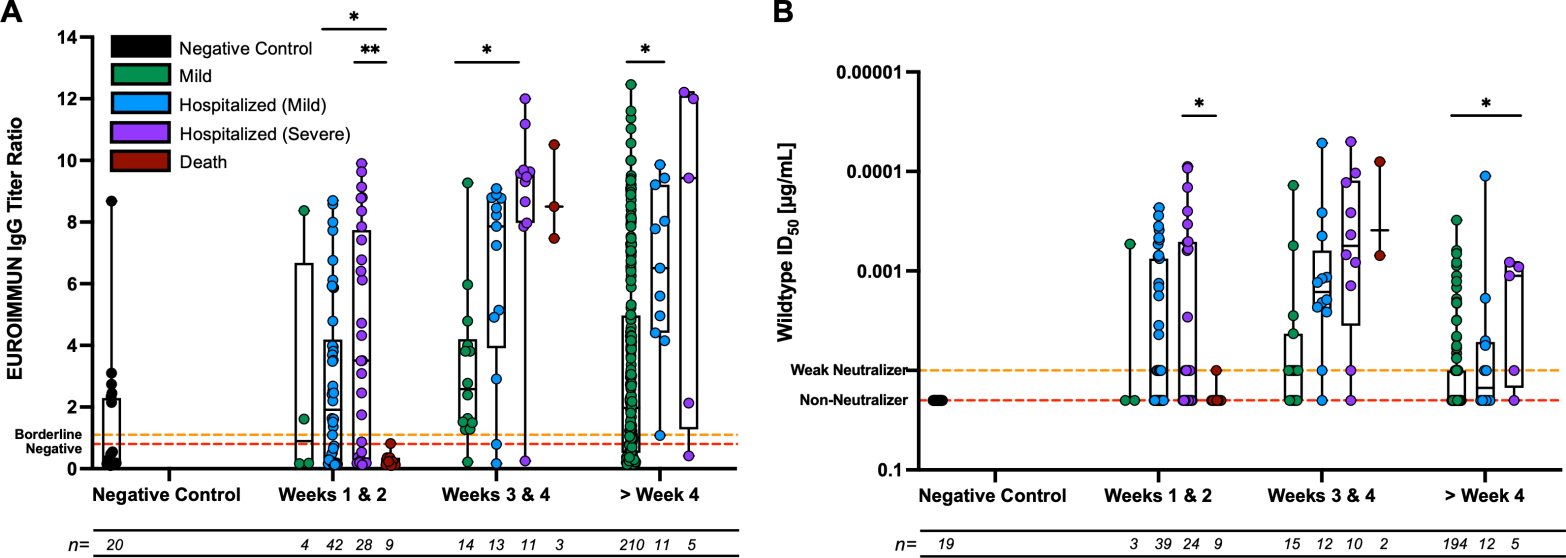
Anti-Spike IgGs and neutralizing antibody responses differ among the clinical spectra of COVID-19 patients depending on time. (**A**) Anti-Spike IgG ratios and (**B**) neutralizing antibodies are shown for mild-symptom patients, hospitalized mild patients, hospitalized severe patients, and deceased patients over time. Box-whisker anti-Spike IgG ratios (**A**) and neutralizing antibody ID_50_ value (**B**) plots illustrate the interquartile range as the box and the minimum and maximum values as the ends of the whiskers. Comparisons between groups were carried out by the ANOVA-Kruskal-Wallis test (*P < 0.05, **P < 0.01, ***P < 0.001, ****P < 0.0001). *N* values plotted and analyzed for each group are shown below the bar. (**A**). The red dotted lines at a ratio of 0.8 signifying those points below this line are negative for anti-Spike IgGs. The orange dotted lines at a ratio of 1.1 signifying the points between the orange and red dotted lines are borderline for anti-Spike IgGs. (**B**) The orange dotted lines at 0.01 µg/mL represent weak neutralizing samples that can neutralize the WT PsV at a dilution of 1:50, but we are unable to fit a proper sigmoidal curve to the data set. The red dotted lines at an ID_50_ value of 0.02 µg/mL represent non-neutralizing samples, which are samples that do not meet any of the criteria to assign an ID_50_ value to the sample.

Several factors including disease severity can influence the strength and breadth of the humoral response (17–21). Many studies have suggested that the humoral response differs in patients with distinct clinical disease progression and infection kinetics (18, 22–28). In our study, we considered different disease severities of COVID-19 as a co-variate for S1 subunit-specific IgG titers (Fig. 2A) or neutralizing antibody titers (Fig. 2B).

In weeks 1 and 2 post-symptom onset, a wide range of EUROIMMUN IgG titer ratios was observed in hospitalized patients with mild and severe disease (Fig. 2A). Samples for patients with mild symptoms or who were deceased revealed low or no anti-S1 IgGs in the serum (Fig. 2A). Notably, a significant difference was seen in anti-S1 IgGs between the hospitalized (mild disease) samples and the deceased patient samples (*P* = 0.0388). Additionally, a significant difference in anti-S1 IgGs was observed between the hospitalized (severe disease) patient samples and the deceased patient samples (*P* = 0.0029) (Fig. 2A). Looking at the ability of the mounted antibodies to neutralize the virus in weeks 1 and 2 PSO, samples from the different disease severity groups exhibited variable neutralizing activity. Higher neutralizing titers were seen in patients who were hospitalized with either mild or severe disease with a median of 0.01 µg/mL (Fig. 2B). In comparison, the majority of mild-symptom patients or deceased patient samples showed no neutralizing antibody activity in weeks 1 and 2 post-symptom onset (Fig. 2B). A significant difference in neutralizing antibody titers was observed between the hospitalized (severe disease) samples and the deceased samples (*P* = 0.0443).

During weeks 3 and 4, hospitalized patients with severe disease and hospitalized patients with mild disease mounted robust S1-IgG-specific antibodies, while those with mild symptoms demonstrated a lower abundance of S1-IgG-specific antibodies (Fig. 2A). Deceased patient samples at weeks 3 and 4 PSO showed higher levels of S1-IgG-specific antibodies compared to samples from deceased patients tested at weeks 1 and 2 PSO and all samples during this timepoint (Fig. 2A). Samples from deceased patients seemed to have only mounted anti-S1 IgGs 3–4 weeks PSO, which was later compared to other groups where anti-S1 IgGs were mounted earlier during weeks 1–2 PSO. A significant difference was seen between mild-symptom samples and hospitalized (severe disease) patients with anti-S1 IgGs (*P* = 0.0280). Assessing antibody neutralization titers during weeks 3–4 PSO, there were higher neutralizing antibody titers in each group during this time-point compared to each group assessed during weeks 1–2. Here again, the trend follows for neutralizing antibody titers where the hospitalized (mild and severe disease) and deceased patient samples had higher neutralizing antibody titers than the mild-symptom patients with a clinical severity score of 1 or 2 (Fig. 2B).

Greater than 4 weeks PSO, a significant difference in EUROIMMUN anti-S1 IgG titers was seen between the mild-symptom samples and hospitalized (mild disease) samples (*P* = 0.0193). A wide range of anti-S1 IgG titers was observed for mild-symptom samples, while hospitalized (mild disease) samples had EUROIMMUN titer ratios between 5 and 10. Similarly, when assessing the neutralizing antibody response in patients greater than 4 weeks PSO, neutralizing antibody titers were higher in all groups other than the mild-symptom patients, and this was consistently true at all the other time points tested. Additionally, overall neutralizing antibody titers in the greater than 4 weeks PSO had lower median values among the different clinical severity categories compared to the other time points. A significant difference was observed between mild-symptom patients and hospitalized patients with severe symptoms greater than 4 weeks post-symptom onset (*P* = 0.0193). These results substantiate the observation that patients experiencing more severe disease are more likely to develop a highly neutralizing antibody response.

### Sustained levels of neutralizing antibodies achieved by hybrid immunity

**Fig 3:**
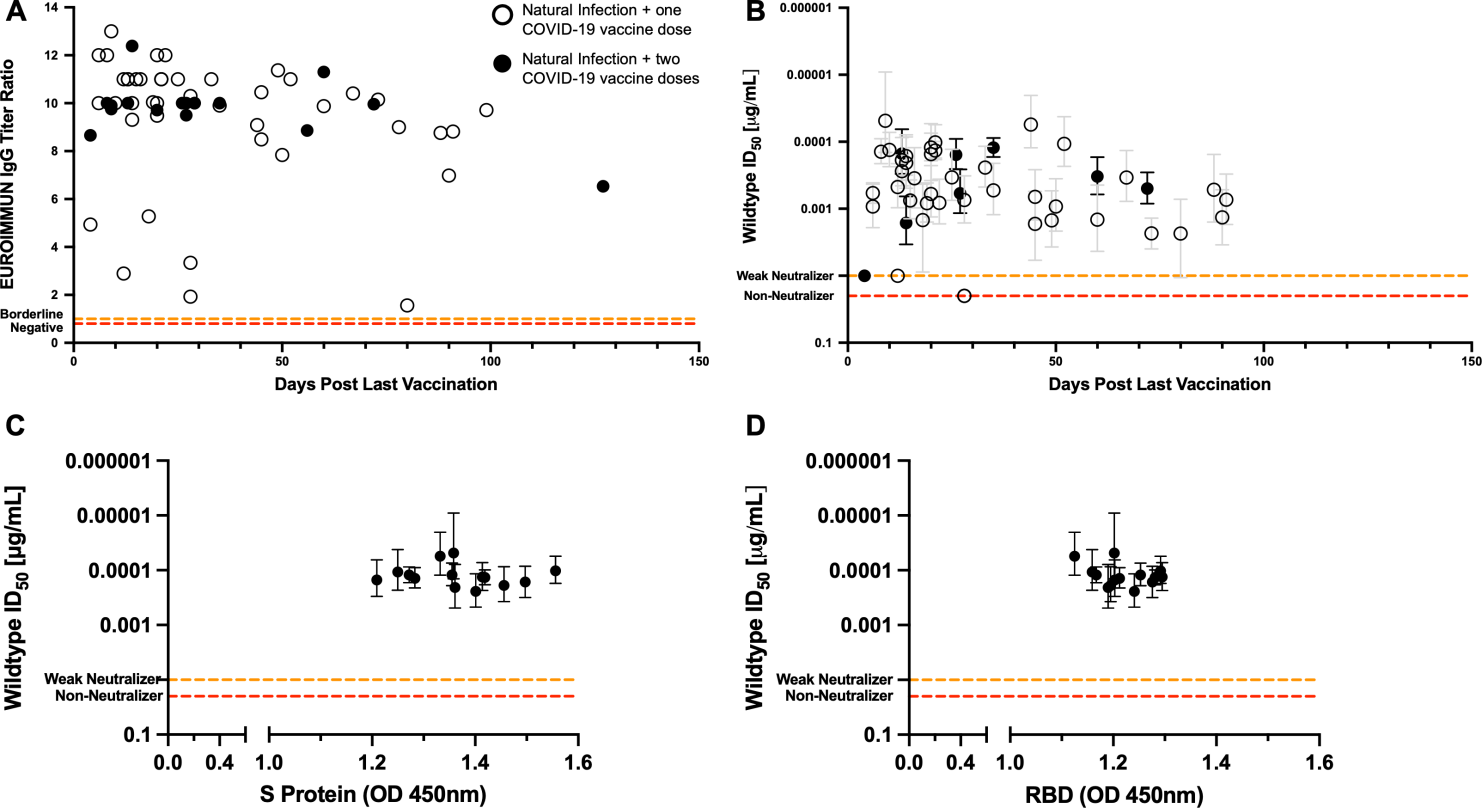
Antibody responses after SARS-CoV-2 vaccination in SARS-CoV-2 recovered individuals over time. (**A**) EUROIMMUN titer ratios of anti-S1 IgG antibodies in naturally infected and vaccinated individuals (cohort B – hybrid immunity) over time. The red dotted lines at a ratio of 0.8 signifying those points below this line are negative for anti-Spike IgGs. The orange dotted lines at a ratio of 1.1 signifying the points between the orange and red dotted lines are borderline for anti-Spike IgGs. (**B**). ID_50_ values of naturally infected and vaccine-induced sera against pseudotyped virus expressing SARS-CoV-2 D614G wild-type spike protein. The bars for each point show the 95% confidence interval. The orange dotted lines at 0.01 µg/mL represent weak neutralizing samples that can neutralize the WT PsV at a dilution of 1:50, but we are unable to fit a proper sigmoidal curve to the data set. The red dotted lines at an ID_50_ value of 0.02 µg/mL represent non-neutralizing samples, which are samples that do not meet any of the criteria to assign an ID_50_ value to the sample. Fourteen of the top neutralizing SARS-CoV-2 serum samples from the cohort and one control sample were tested by ELISA at a dilution of 1:50 dilution for the presence of SARS-CoV-2 total spike protein-specific (**C**) and RBD-specific (**D**) total antibodies. The bars for each point show the 95% confidence interval.

With the development and approval of SARS-CoV-2 vaccines, it was pertinent to understand the antibody immune response elicited by vaccination, particularly in the context of previous infection. Therefore, we evaluated the anti-S1 IgG response and neutralizing antibody titers in cohort B – hybrid immunity (naturally infected and then subsequently vaccinated). All hybrid patient samples were positive for anti-S1 IgGs in the sera up to 127 days post-vaccination, and a wide range of anti-S1 IgG titers was observed (Fig. 3A). In the same cohort, individuals who had two doses of the vaccine showed stable levels of anti-S1 IgGs (Fig. 3A). Hybrid-immunity samples strongly neutralized the wild-type (D614G) spike protein, except for two cases that showed no neutralization and another two cases where they were classified as a weak neutralizer (Fig. 3B). Hybrid samples that mounted no or weak neutralizing antibodies may be, in part, due to the early timepoint at which the samples were tested post-immunization; indeed, the two weak neutralizers were taken 4 to 12 days post-vaccination, while the two non-neutralizing cases were from patient samples taken 28 days post-vaccination. The second dose of the vaccine did not seem to increase neutralizing antibody titers but instead helped sustain the neutralizing antibody titers at similar levels as the first dose of the vaccine. Testing the top neutralizing samples in this cohort (*n* = 14), antibodies present in the serum bound to both the entire wild-type spike protein (Fig. 3C) and the RBD of the virus (Fig. 3D). Additionally, in this cohort, the titers of neutralizing antibodies were positively correlated with the IgG titers with an R^2^ value of 0.62 (Fig. S2A).

### Hybrid immunity elicits stronger responses compared to natural infection alone

**Fig 4:**
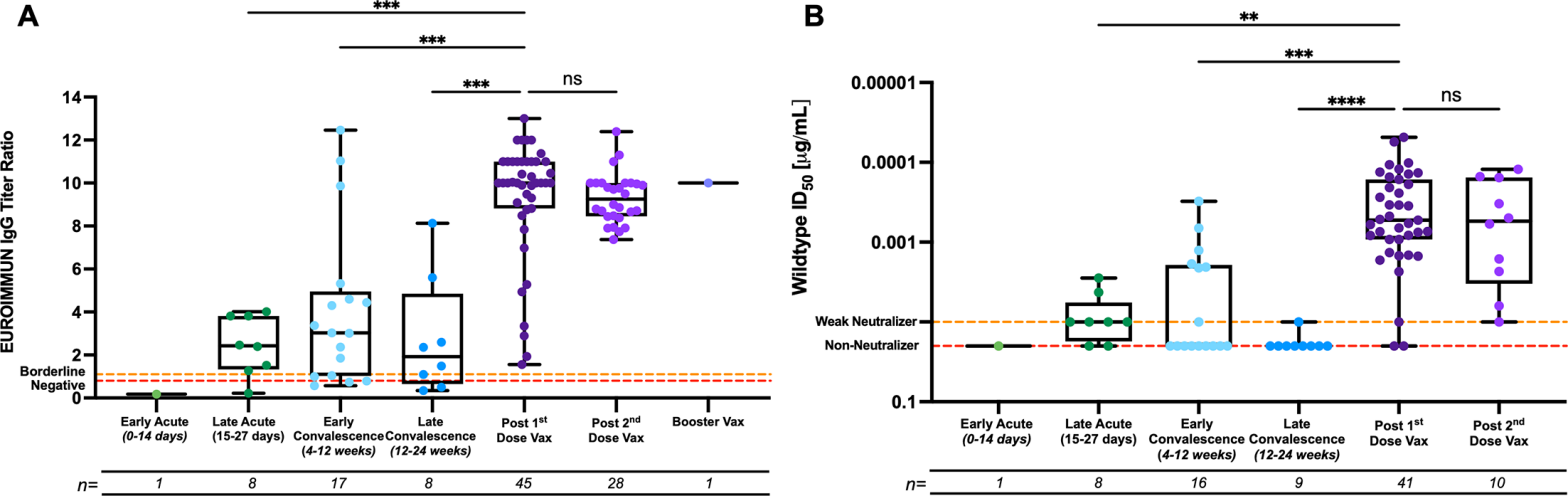
Comparison of antibody responses elicited by natural infection and hybrid immunity. (**A**) Anti-S1 IgG antibody titer ratios of naturally infected study participants before and after SARS-CoV-2 vaccination were measured using the EUROIMMUN assay. Sera were collected from study participants during infection but pre-vaccination, either in the early acute (0–14 days) (*n* = 1), late acute (15–27 days) (*n* = 7), early convalescent (4–12 weeks) (*n* = 16), or late convalescent (12–24 weeks) (*n* = 8) phases of the infection and subsequently after the first dose (*n* = 45), the second dose (*n* = 28), or the booster dose (*n* = 1) of the SARS-CoV-2 vaccine. The red dotted lines at a ratio of 0.8 signifying those points below this line are negative for anti-Spike IgGs. The orange dotted lines at a ratio of 1.1 signifying the points between the orange and red dotted lines are borderline for anti-Spike IgGs. (**B**) Neutralizing antibody titers of naturally infected study participants without or with a previous history of SARS-CoV-2 vaccination were measured on the D614G pseudotyped virus. Sera were collected from study participants during infection but pre-vaccination, either in the early acute (0–14 days) (*n* = 1), late acute (15–27 days) (*n* = 8), early convalescent (4–12 weeks) (*n* = 16), or late convalescent (12–24 weeks) (*n* = 9) phases of the infection and subsequently after the first dose (*n* = 40) or the second dose (*n* = 10) of the SARS-CoV-2 vaccine. The orange dotted lines at 0.01 µg/mL represent weak neutralizing samples that can neutralize the WT PsV at a dilution of 1:50, but we are unable to fit a proper sigmoidal curve to the data set. The red dotted lines at an ID_50_ value of 0.02 µg/mL represent non-neutralizing samples, which are samples that do not meet any of the criteria to assign an ID_50_ value to the sample. COVID-19 history was determined by symptoms and a PCR+ test or serology. Box-whisker anti-Spike IgG ratios (**A**) and neutralizing antibody ID_50_ value plots (**B**) illustrate the interquartile range as the box and the minimum and maximum values as the ends of the whiskers. Comparisons between groups were carried out by the ANOVA-Kruskal-Wallis test (*P < 0.05, **P < 0.01, ***P < 0.001, ****P < 0.0001). *N* values plotted and analyzed for each group are shown above the bar.

In the subset of patients who enrolled in our study after natural infection and followed up with a subsequent visit after vaccination, 79% of individuals had detectable levels of anti-S1 IgGs before vaccination, and the remainder (7/34 or 21%) tested negative for anti-S1 IgG antibodies before vaccination; the latter ranged from early acute to late convalescent phase (Fig. 4A). Correspondingly, only 24% of these samples were positive for neutralizing antibodies against the wild-type D614G strain, and 18% mounted weak neutralizing antibodies(Fig. 4B).

The antibody responses, both anti-S1 IgG titers and neutralizing antibody titers, significantly increased after the first dose of the vaccine. Indeed, a significant difference was observed in S1-binding IgGs among the late acute (*P* = 0.0002), early convalescent (*P* = 0.0002), and late convalescent phases of the infection (*P* = 0.0003), compared to samples derived after the first dose of a SARS-CoV-2 vaccine (Fig. 4A). No additional increase or a significant difference in antibody levels was detected after a second dose of the vaccine (Fig. 4A). The same trend was observed when assessing the neutralizing antibody response where individuals had a robust neutralizing antibody response after primary immunization, with no further increase in neutralization titers against the wild-type D614G strain after the second dose of the vaccine (Fig. 4B). All patient samples in this subset were positive for anti-S1 IgGs after vaccination, although among these, one person mounted weak neutralizing antibodies and two individuals did not mount neutralizing antibodies against the D614G pseudotyped virus after one dose of the vaccine (Fig. 4B), as described above. Furthermore, after two doses of the vaccine, all but one sample was able to induce neutralizing antibodies against the D614G pseudotyped virus. Like the anti-S1 IgG-binding data, a significant difference was observed in neutralizing antibodies among the late acute (*P* = 0.0176), early convalescent (*P* = 0.0005), and late convalescent phases of the infection (*P* < 0.0001), compared to the first dose of the SARS-CoV-2 vaccine (Fig. 4B). Additionally, there was no significant difference observed between the neutralizing antibodies mounted after the first and second doses of the SARS-CoV-2 vaccine.

### Hybrid immunity elicits neutralizing antibody responses against variants of concern

**Fig 5:**
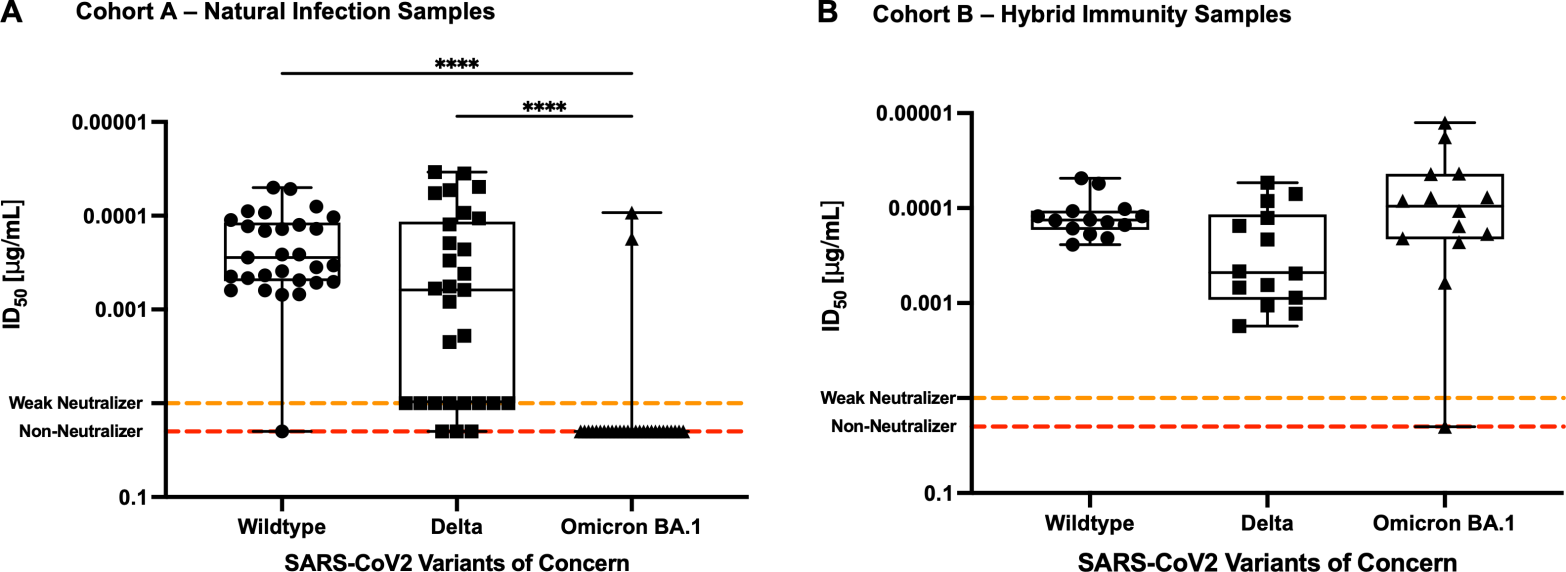
Antibody neutralization persists against variants of concern in individuals with hybrid immunity. Neutralizing antibody activity against the wild-type (D614G), Delta, and Omicron BA.1 pseudotyped virus variants in serum from cohort A – naturally infected patients (*n* = 29) (**A**) and from cohort B – hybrid-immunity samples (*n* = 14) (**B**). The orange dotted lines at 0.01 µg/mL represent weak neutralizing samples that can neutralize the WT PsV at a dilution of 1:50, but we are unable to fit a proper sigmoidal curve to the data set. The red dotted lines at an ID_50_ value of 0.02 µg/mL represent non-neutralizing samples, which are samples that do not meet any of the criteria to assign an ID_50_ value to the sample. Comparisons between groups were carried out by the ANOVA-Kruskal-Wallis test (*P < 0.05, **P < 0.01, ***P < 0.001, ****P < 0.0001).

The top 43 neutralizing patient serum samples against the SARS-CoV-2 ancestral wild-type (D614G) variant were tested against the Delta and Omicron BA.1 VOCs pseudotyped viruses. These two variants of concern were chosen to test our samples because these SARS-CoV-2 strains quickly dominated all other circulating strains worldwide during the time of this study. In this smaller subset of our cohort, *n* = 29 patient samples were derived from cohort A – natural infection only (7–360 days post-infection, with a median of 18 days), and the other *n* = 14 patient samples were derived from cohort B – patients with hybrid immunity (8–52 days post-vaccination, with a median of 17 days) (Table 1). All samples from cohort A neutralized the wild-type D614G PsV strain (Fig. 5A). Against the Delta PsV strain, there was a wider range of ID_50_ values: 62% of the cohort was positive for neutralizing antibodies, 28% of the cohort was weak neutralizers, and 10% of the cohort was non-neutralizers (Fig. 5A). Of note, only 10% (*n* = 2) of cohort A was positive for Omicron BA.1-neutralizing antibodies, while the rest of the patient samples were unable to neutralize the Omicron BA.1 PsV strain (Fig. 5A). In contrast, in the hybrid cohort (cohort B), all samples neutralized the wild-type (D641G) and the Delta PsV strains, albeit with a wider range of ID_50_ values against the Delta PsV compared to the wild-type PsV (Fig. 5B). Strikingly, all samples but one neutralized the Omicron BA.1 PsV strain (Fig. 5B). These data highlight how antibodies elicited by hybrid immunity are more resilient against different VOCs compared to neutralizing antibodies mounted from a single natural infection event.

## DISCUSSION

Antibodies play a crucial role in establishing protective immunity against emerging viral infections. It is essential to determine their longevity and robustness across various demographics, as this information is pivotal in assessing seroprevalence within communities and in formulating effective vaccination strategies against the illnesses targeted by specific vaccines. In several studies, IgG and neutralizing antibody levels are significantly associated with the risk of SARS-CoV-2 infection and could serve as correlates of protection for infection and intensity of symptomatic disease (29, 30). Therefore, in this study, we examined the kinetics of antibody levels over a period of 1 year post-COVID-19 symptom onset in a cohort of patients herein described.

We observed peak anti-S1 IgG levels approximately 3–4 weeks following infection. Antibody levels were stable and sustained up to 3 months post-infection. Eventually, decay was observed in the levels of anti-S1 IgGs over the 12-month period, where anti-S1 IgGs persisted at detectable levels in some patients beyond the early convalescent phase of the infection, as has been described in other studies (30). The concentration of IgG antibodies to spike and RBD was also highly correlated with pseudovirus-neutralizing antibody titers. We found long-lasting neutralizing antibody titers that peaked during the late acute to early convalescent phase of the infection (between 1 and 3 months) in most individuals. During the latter half of the early convalescent phase of the infection, decay was observed in this cohort. In this regard, neutralizing antibodies were sustained at low titers among some individuals, while the majority of patients completely lost neutralizing antibody titers.

The humoral response is influenced by several factors, including clinical disease progression (9, 31–33). Our analysis shows that the antibody response is significantly associated with disease severity. Hospitalized patients (with mild or severe symptoms) exhibited higher anti-S1 IgG titers and neutralization titers compared to patients who were asymptomatic or had milder symptoms not requiring hospitalization. It is noteworthy that within each disease severity classification at the different time points, a wide range of responses was observed; however, a higher median of anti-S1 IgG and neutralizing antibodies was observed in more severe cases during the earlier time points. This association has been independently reported by other groups and can be partly explained by the possibility that uncontrolled viral spread, leading to increased pathology, exacerbated inflammation, and an increase in viral antigen load favors a strong humoral response (33–36). Additionally, comorbidities have been shown in the literature to contribute to a wide range of antibody responses (37, 38). Individuals with mild or asymptomatic infection may also serorevert more quickly than symptomatic individuals (30). The gradation of responses by disease severity has also been found in other infections, including SARS-CoV-1 and MERS-CoV infection (39).

Studies assessing the longevity of the humoral response against SARS-CoV-2 vary widely depending on sampling and detection assay strategies: some groups have observed stable antibody levels for several months (27, 30, 34), while other reports showed a rapid decline of anti-SARS-CoV-2 IgGs 3 months post-symptom onset (26, 40). Our results expand on previous findings comparing neutralization levels with antigen-specific responses over a long follow-up period. We observed that the decline in IgG titers and neutralization often stabilized at different levels later into convalescence. Decreasing IgG titers and neutralizing antibodies eventually plateaued for some patients while others may have seroreverted, and anti-S1 IgGs and neutralizing antibodies were undetectable at later time-points post-symptom onset. While our results reveal widely different magnitudes of initial responses and decay in neutralizing antibody titers, approximately 47% of the samples had detectable Spike IgGs, however, with only 7% of the samples possessing strong neutralizing responses and 14% possessing weak neutralizing responses more than 6 months PSO. These data highlight that a discrepancy between antibody-binding titers and neutralizing titers might emerge particularly as antibody titers wane. The importance of these observations is that following recovery from natural infection, neutralizing antibodies may persist, albeit at low levels, and may occasionally act as the first line of defense against future encounters of SARS-CoV-2 and possibly related human coronaviruses (41).

Some COVID-29 vaccines were shown to elicit levels of neutralizing antibodies that are comparable to those observed in naturally infected individuals; however, this was dependent on the vaccine type (7, 42, 43). Our study provides quantitative data showing that anti-S1 IgGs and long-lasting neutralizing antibody responses induced through natural infection can be significantly boosted after immunization, which has also been observed by others (44–46). We found that up to 3 months after the first and second doses of the SARS-CoV-2 vaccine, individuals had sustained levels of anti-S1 IgGs and neutralizing antibodies. Previous studies have shown that neutralizing antibody levels decrease over time after initial vaccination with the COVID-19 vaccines and show a substantial decrease by 6 to 8 months, which is a timeline not covered in our study (47, 48). Moreover, we noted that approximately 8% of vaccinated individuals did not seroconvert or mount weak neutralizing antibody titers, which has also been reported by others (49, 50). Our serological data are also consistent with several other studies that indicate robust boosting of antibody responses in SARS-CoV-2 recovered subjects after the first vaccine dose but little benefit to SARS-CoV-2-specific antibody levels and neutralizing antibodies after the second vaccine dose (45, 51–56). More samples post-vaccination follow-up collected with a wider range of days post-vaccination may help solidify these results more. With the widespread use of SARS-CoV-2 vaccines, it is essential to understand changes in seropositivity, neutralizing antibody levels, and the overall humoral response over time to guide future vaccination strategies.

Waning neutralizing antibody titers and the emergence of more neutralization-resistant SARS-CoV-2 variants have resulted in enhanced numbers of breakthrough infections globally (57–60). In our serological study, we found that Delta and Omicron BA.5 were less prone to neutralization compared to wild-type D614G. In patients who only acquired natural immunity, we observed profound escape of the Omicron variant where 90% of the samples did not mount neutralizing antibodies against Omicron BA.5. The data indicate that convalescent patients infected by the wild-type D614G strain would be particularly susceptible to being reinfected by other strains of SARS-CoV-2 despite these patients mounting the highest neutralizing antibody titers against wild-type D614G in our cohort. The neutralizing antibody response differs quite strikingly in the hybrid cohort, who were also originally presumed to have been infected with the wild-type D614G virus based on the timing of infection. All individuals in this cohort were able to protect against Delta, albeit with lower neutralizing antibody titers compared to wild-type D614G. Additionally, all but one individual mounted neutralizing antibodies against Omicron BA.5. Our observations showcase the difference in the quality of the antibody response mounted after hybrid immunity is achieved, where even lower neutralizing antibody titers can be resilient against VOCs. Higher antibody responses were observed among individuals with more severe COVID-19 symptoms, and when coupled with vaccination, they explain the durable and resilient protection against Delta and Omicron BA.5. Future studies will be required to determine whether this resilient *in vitro* neutralization against VOCs observed after hybrid immunity in individuals with more severe COVID-19 symptoms is associated with protection from subsequent infections when VOCs are circulating.

Our study has certain limitations. In this regard, although some individuals were unable to continue in the study and the timing of sample collection varied for others, our findings still provide valuable insights. We were able to gather valuable data from a combination of both cross-sectional and longitudinal samples. The median ages of the naturally infected-only cohort (cohort A) and the hybrid cohort (cohort B) were 42 and 43 years, respectively; thus, the results might not be generalizable to older adults or children. Despite this, in our cohort, we have assessed children 0–12 years of age and individuals more than 60 years of age and did not see obvious differences in the decay rate and kinetics of the antibody response. Further assessment of the effect of age, sex, gender, ethnicity, obesity, and other comorbidities in the natural infection and vaccine response is warranted with more statistical power. Another limitation is that virus lineage information was not available for patients in this study. In all cases, they were infected between March 2020 and May 2021, before the emergence of the Delta and Omicron variants of concern in Canada, and so the Wuhan strain infection is assumed. Neutralizing antibody responses were assessed against the wild-type (D614G), Delta, and Omicron BA.5 strains only, therefore precluding insights related to more current variants of concern such as Omicron BQ and XBB.

Overall, our study showed that anti-Spike IgGs and neutralizing antibody dynamics vary greatly among individuals with COVID-19, as reflected by peak antibody levels, rates of waning, and longevity of the antibody responses. Additionally, we found an association between robust antibody responses and severe COVID-19 clinical symptoms during the first-month post-symptom onset. SARS-CoV-2-experienced individuals had a robust boost in anti-S1 IgGs and neutralizing antibodies subsequently after the first dose of the SARS-CoV-2 vaccine; however, there was no change in the antibody response after the administration of the second dose of the vaccine. Most importantly, neutralizing antibodies elicited by natural infection alone were largely ineffective against emerging variants of concern, in contrast to those elicited in hybrid immunity that were more resilient. Further studies will be required to continue the evaluation of the durability and resilience against continuously emerging variants of concern for neutralizing antibodies induced by subsequent vaccine doses. As SARS-CoV-2 variants continue to emerge, understanding the interplay between infection, vaccine durability, and virus evolution will be critical for revising vaccination strategies and formations to prevent severe disease and hospitalization in the population.

## MATERIALS AND METHODS

### Study design and participants

Persons with a diagnosis of COVID-19 were prospectively enrolled and followed with a plan for serial sampling during the acute and convalescent phases of COVID-19. They were eligible for enrollment if COVID-19 was diagnosed by PCR testing or rapid antigen testing on nasopharyngeal swabs in the presence of respiratory or other symptoms consistent with COVID-19. Controls were collected from the same participant pool and were deemed naïve controls if they were symptom free, tested negative in either a PCR or a rapid antigen nasopharyngeal swab test, or were negative through serological testing. Samples were collected at The Hospital for Sick Children, Mount Sinai Hospital, and North York General Hospital in Toronto, Ontario, Canada.

### Clinical assessments, data, and sample collection

Following the prospective enrollment of patients with confirmed COVID-19, medical records were reviewed to obtain pertinent clinical and related data. Collected data included date of birth, age at enrollment, sex, COVID-19 PCR status, date of symptom onset or date of positive PCR if asymptomatic and tested, clinical severity score, visit date, date of vaccines dose(s), and type of vaccine. After centrifugation for collection of serum, samples were stored at −80°C.

#### Clinical severity

Clinical severity scores were assigned to patients based on their symptoms, and this scoring scheme was based on the criteria initially developed for the WHO (16). Patients with a clinical severity score of 1 or 2 were considered to have “mild symptoms,” a score of 3 or 4 was “hospitalized with mild disease,” a score of 5, 6, or 7 were patients “hospitalized with severe disease,” and a score of 8 were patients who were “deceased.”

#### Hybrid immunity

Immunity provided by a combination of SARS-CoV-2 infection and SARS-CoV-2 vaccination. In this context, at least one episode of infection would have preceded vaccination.

### Laboratory procedures

#### Serologic testing

##### EUROIMMUN anti-S1 IgG ELISA

SARS-CoV-2 IgG antibodies were determined using the EUROIMMUN assay, which is an approved enzyme-linked immunoassay (EUROMMUN, Lubeck, Germany) (61). This is a semi-quantitative assay that targets the recombinant S1 protein of SARS-CoV-2. The interpretation of results was based on the signal-to-cutoff ratios of <0.8 being reported as negative, ≥0.8 to <1.1 as borderline, and ≥1.1 as positive. The sensitivity of the assay is reported to be 94.4% with a specificity of 99.6% among persons who are >10 days after the onset of symptoms (62, 63).

### Viral inactivation

Inactivation of potential infectious viruses in serum was performed by heating the previously frozen samples to 60°C for 30 minutes. Heat-inactivated serum samples were then stored at −80°C until further use in downstream immunological assays.

### Cell lines and maintenance

FreeStyle 293-F Cells (FreeStyle 293-F cells, Thermo Fisher Scientific; HEK 293S, GnT I^-/-^ cells, American Type Culture Collection [ATCC]) were cultured in suspension in FreeStyle 293 Expression Medium (Gibco) and were maintained at 37°C in a Multitron Pro Shaker (Infors HT) with 8% CO_2_ and 70% humidity, on a shaker platform rotating at 130 rpm.

For pseudovirus neutralization assays, human embryonic kidney (HEK) 293 cells containing the SV40 T-antigen (HEK293T) (ATCC) and HEK293T-ACE2 cells (BEI NR2511) were cultured in Dulbecco’s Modified Eagle’s Medium High Glucose (DMEM; Gibco) supplemented with 10% heat-inactivated fetal bovine serum (FBS; Gibco), 2.5% HEPES (Gibco), and 0.5% gentamicin (Thermo Fisher) in a 37°C, 5% CO_2_ incubator. All cells were negative for mycoplasma.

### Expression and purification of recombinant SARS-CoV-2 spike (S) protein and RBD

FreeStyle 293-F cells were split to a density of 0.8 × 10^6^ cells/mL at least 1 hour before transfection. Cells were transfected using FectoPRO Reagent (Polyplus) following the manufacturer’s instructions at a 1:1 DNA to FectoPRO ratio. Plasmid DNA (50 µg) encoding the SARS-CoV-2 Spike (S) protein or RBD was used per 200 mL of cell culture. Recombinant proteins were purified by affinity chromatography via a HisTrap Ni-NTA column (Cytiva) and were eluted using 20 mM Tris pH 8.0, 500 mM imidazole buffer. Subsequent size exclusion chromatography was performed in 20 mM Tris, pH 8.0, 150 mM NaCl on a Superdex 200 Increase column (Cytiva). All purified proteins were validated for integrity and purity via sodium dodecyl sulfate-polyacrylamide gel electrophoresis (SDS-PAGE) and were stored at −80°C until use.

### Anti-RBD and anti-spike protein enzyme-linked immunosorbent assay (ELISA)

The ELISA procedure in this study was modified from a protocol published by Burn Aschner et al. (64). Briefly, Nickel-coated 96-well plates (Pierce, Thermo Fisher Scientific) were coated overnight with 50 µL per well of His-tagged SARS-CoV-2 RBD wild-type (D614G) protein or His-tagged SARS-CoV-2 wild-type (D614G) spike (S) protein in Tris-buffered saline (TBS) (20 mM Tris, pH 8.0, 150 mM NaCl) at 4°C. Plates were washed one time with TBS + 0.05% Tween 20 (TBS-T) and were subsequently blocked with TBS + 5% bovine serum albumin for 1 hour at room temperature. Plates were washed one time with TBS-T, incubated with serum diluted 1:50, and subsequently serially diluted 1 in 4 for 1 hour at room temperature. Plates were washed two times with TBS-T, and bound antibodies were detected using a horseradish peroxidase-conjugated anti-human Fab secondary antibody (Abcam, 87422) for 1 hour at room temperature. Plates were washed three times with TBS-T and were developed using 3,3′,5,5′-Tetramethylbenzidine (TMB) substrate reagent set (BD Biosciences) following the manufacturer’s instructions. Reactions were stopped by the addition of 2N HCl. Data were collected using a Synergy Neo2 Multi-Mode Assay Microplate Reader (Biotek Instruments).

### SARS-CoV-2 pseudovirus (PsV) production

The SARS-CoV-2 PsV production and neutralization assay procedure in this study was modified from a protocol published by Burn Aschner et al. (64). PsVs were produced using a lentiviral vector backbone, as previously described by Corman et al. (65). Briefly, SARS-CoV-2 PsVs were generated by transient co-transfection of HEK293T cells with a lentiviral backbone encoding luciferase reporter gene (BEI NR52516), plasmids encoding Gag-Pol (BEI NR52517), Tat (BEI NR52518), Rev (BEI NR52519), and a plasmid expressing SARS-CoV-2 spike protein (BEI NR52310) using the BioT transfection reagent (Bioland Scientific) according to the manufacturer’s directions. Transfected cells were incubated at 37°C for 24 hours, followed by the addition of 5 mM of sodium butyrate. Transfected cells were further incubated for an additional of 24 to 30 hours at 30°C. SARS-CoV-2 Spike mutant D614G was kindly provided by D. R. Burton (Scripps Research) and was used to generate the SARS-CoV-2 wild-type PsV. Delta (B.1.617.2) SARS-CoV-2 PsV variant was generated by substituting the wild-type spike plasmid with the respective VOC spike using plasmids kindly provided by David Ho (Columbia). Omicron (BA.5) was generated using a spike plasmid synthesized at GeneArt (Invitrogen). PsVs were harvested, filtered through 0.45 µm sterile filters, and concentrated using 100 K Amicon filters (Millipore Sigma).

### SARS-CoV-2 PsV neutralization assay

Neutralization assays were performed using 293T-ACE2 cells (BEI NR52511) as previously described with a few modifications (66, 67). Briefly, 96-well plates were coated with poly-L-Lysine (Sigma-Aldrich), and cells were seeded at a density of 10,000 cells/well in 100 µL. The following day, serial dilutions of the heat-inactivated human sera or control IgG antibodies in duplicates were incubated with the PsV for 1 hour at 37°C. Cell culture medium was then replaced with the PsV-sera or PsV-IgG mixture supplemented with 10 µg/mL polybrene (Sigma-Aldrich). Cells were incubated for 48 hours at 37°C, before the addition of 50 µL of Britelite plus reagent (PerkinElmer) for 2 minutes. Supernatants were transferred to 96-well white plates (Sigma-Aldrich), and luminescence in relative light units (RLUs) was measured using a Synergy Neo2 Multi-Mode Assay Microplate Reader (Biotek Instruments). Absorbance data were converted to percent inhibition using the following formula:

Percent inhibition = (1 – [OD sample – OD min] / [OD max – OD min]) × 100%.

Percent inhibition was plotted, and data were fitted using the non-linear 4PL regression, constrained at top = 100% and bottom = 0% in GraphPad Prism version 10.02 to determine the ID_50_ values of each sample. ID_50_ values were assigned to samples based on three criteria:

1. The R squared value is above 0.5 when the neutralization curves are fit with a sigmoidal fit.
2. The log ID_50_ in the 95% confidence interval is between three log units.
3. The 1:50 serum dilution (i.e., the lowest dilution in the series) neutralizes above 50%.

For neutralizing samples, all three criteria are met, which means an ID_50_ value could be confidently calculated for the sample. Samples are classified as weakly neutralizing if criteria 3 is met; however, criteria 1 or 2 is not met. An ID_50_ value cannot be confidently assigned to this sample because the sample is still neutralizing at the lowest dilution used, thus we assigned an ID_50_ value of 0.01 to these samples. Lastly, non-neutralizers are samples where all three criteria are not met; we assigned these samples an ID_50_ value of >0.02 as the ID_50_ is below the limit of detection for this assay. Plotted ID_50_ values are the average of at least two to three biological replicates with two technical replicates each when performed.

### Statistical analysis

Statistical analyses were performed using GraphPad Prism version 10.02 and R (Foundation for Statistical Computing, Vienna, version 4.2.2). We reported participant characteristics using median (with interquartile range [IQR]) for continuous variables, and counts (with percentage) for categorical variables. Wherever applicable, the normality of the data was determined by the Shapiro-Wilk test to select the most appropriate statistical test. For comparison of two groups, the Student’s *t*-test (normal distribution) or Mann-Whitney (non-normal distribution) was performed. For comparison of more than two groups, a one-way analysis of variance (ANOVA) (normal distribution) or a Kruskal-Wallis followed by Dunn’s multiple comparisons test (non-normal distribution) was performed. Locally estimated scatterplot smoothing (LOESS) non-parametric function was used to visualize trends in EUROIMMUN anti-S1 IgG levels and neutralizing antibody levels over days post-symptom onset. All LOESS analyses were completed using R (Version 4.2.2) within Rstudio (Version 2023.03.01+446) and were visualized using ggplot2. Statistical significances are indicated in the figures by asterisks as follows: *P < 0.05, **P < 0.01, ***P < 0.001, or ****P < 0.0001. Across all LOESS analyses, a span of 0.5 or 0.6 with a 95% confidence interval was applied. Correlations were assessed using the Pearson correlation test to assess the linear relationship between variables, specifically to look at the correlation between the EUROIMMUN anti-S1 IgG titer ratios and the neutralizing antibody titers (ID_50_ values). The goodness of fit for linear regression analyses was reported as the coefficient of determination, R^2^.

## SUPPLEMENTAL MATERIAL

Figure S1 and S2 spectrum.01333-24-s0001.docx Antibody responses from naturally infected patients wane over time and the correlation between anti-S1 IgGS and neutralizing antibodies.

ASM does not own the copyrights to Supplemental Material that may be linked to, or accessed through, an article. The authors have granted ASM a non-exclusive, world-wide license to publish the Supplemental Material files. Please contact the corresponding author directly for reuse.

## References

[1] WHO Coronavirus (COVID-19) dashboard with vaccination data. Available from: https://covid19.who.int/?mapFilter=deaths

[2] Sette A, Crotty S. 2021. Adaptive immunity to SARS-CoV-2 and COVID-19. Cell 184:861–880. doi:10.1016/j.cell.2021.01.007 33497610 PMC7803150

[3] Crotty S. 2021. Hybrid immunity. Science 372:1392–1393. doi:10.1126/science.abj2258

[4] Regev-Yochay G, Lustig Y, Joseph G, Gilboa M, Barda N, Gens I, Indenbaum V, Halpern O, Katz-Likvornik S, Levin T, Kanaaneh Y, Asraf K, Amit S, Rubin C, Ziv A, Koren R, Mandelboim M, Tokayer NH, Meltzer L, Doolman R, Mendelson E, Alroy-Preis S, Kreiss Y. 2023. Correlates of protection against COVID-19 infection and intensity of symptomatic disease in vaccinated individuals exposed to SARS-CoV-2 in households in Israel (ICoFS): a prospective cohort study. Lancet Microbe 4:e309–e318. doi:10.1016/S2666-5247(23)00012-5 36963419 PMC10030121

[5] Jackson CB, Farzan M, Chen B, Choe H. 2022. Mechanisms of SARS-CoV-2 entry into cells. Nat Rev Mol Cell Biol 23:3–20. doi:10.1038/s41580-021-00418-x 34611326 PMC8491763

[6] Walls AC, Park Y-J, Tortorici MA, Wall A, McGuire AT, Veesler D. 2020. Structure, function, and antigenicity of the SARS-CoV-2 spike glycoprotein. Cell 181:281–292. doi:10.1016/j.cell.2020.02.058 32155444 PMC7102599

[7] Yaugel-Novoa M, Bourlet T, Paul S. 2022. Role of the humoral immune response during COVID-19: guilty or not guilty? Mucosal Immunol 15:1170–1180. doi:10.1038/s41385-022-00569-w 36195658 PMC9530436

[8] Wei J, Matthews PC, Stoesser N, Maddox T, Lorenzi L, Studley R, Bell JI, Newton JN, Farrar J, Diamond I, Rourke E, Howarth A, Marsden BD, Hoosdally S, Jones EY, Stuart DI, Crook DW, Peto TEA, Pouwels KB, Walker AS, Eyre DW, COVID-19 Infection Survey team. 2021. Anti-spike antibody response to natural SARS-CoV-2 infection in the general population. Nat Commun 12:6250. doi:10.1038/s41467-021-26479-2 34716320 PMC8556331

[9] Wajnberg A, Amanat F, Firpo A, Altman DR, Bailey MJ, Mansour M, McMahon M, Meade P, Mendu DR, Muellers K, Stadlbauer D, Stone K, Strohmeier S, Simon V, Aberg J, Reich DL, Krammer F, Cordon-Cardo C. 2020. Robust neutralizing antibodies to SARS-CoV-2 infection persist for months. Science 370:1227–1230. doi:10.1126/science.abd7728 33115920 PMC7810037

[10] Guan W-J, Ni Z-Y, Hu Y, Liang W-H, Ou C-Q, He J-X, Liu L, Shan H, Lei C-L, Hui DSC, et al.. 2020. Clinical characteristics of coronavirus disease 2019 in China. N Engl J Med 382:1708–1720. doi:10.1056/NEJMoa2002032 32109013 PMC7092819

[11] Goyal P, Choi JJ, Pinheiro LC, Schenck EJ, Chen R, Jabri A, Satlin MJ, Campion TR Jr, Nahid M, Ringel JB, Hoffman KL, Alshak MN, Li HA, Wehmeyer GT, Rajan M, Reshetnyak E, Hupert N, Horn EM, Martinez FJ, Gulick RM, Safford MM. 2020. Clinical characteristics of Covid-19 in New York City. N Engl J Med 382:2372–2374. doi:10.1056/NEJMc2010419 32302078 PMC7182018

[12] Huang C, Wang Y, Li X, Ren L, Zhao J, Hu Y, Zhang L, Fan G, Xu J, Gu X, et al.. 2020. Clinical features of patients infected with 2019 novel coronavirus in Wuhan, China. Lancet 395:497–506. doi:10.1016/S0140-6736(20)30183-5 31986264 PMC7159299

[13] Jeffery-Smith A, Rowland TAJ, Patel M, Whitaker H, Iyanger N, Williams SV, Giddings R, Thompson L, Zavala M, Aiano F, Ellis J, Lackenby A, Höschler K, Brown K, Ramsay ME, Gopal R, Chow JY, Ladhani SN, Zambon M. 2021. Reinfection with new variants of SARS-CoV-2 after natural infection: a prospective observational cohort in 13 care homes in England. Lancet Healthy Longev 2:e811–e819. doi:10.1016/S2666-7568(21)00253-1 34873592 PMC8635459

[14] Liu C, Ginn HM, Dejnirattisai W, Supasa P, Wang B, Tuekprakhon A, Nutalai R, Zhou D, Mentzer AJ, Zhao Y, et al.. 2021. Reduced neutralization of SARS-CoV-2 B.1.617 by vaccine and convalescent serum. Cell 184:4220–4236. doi:10.1016/j.cell.2021.06.020 34242578 PMC8218332

[15] Zani A, Caccuri F, Messali S, Bonfanti C, Caruso A. 2021. Serosurvey in BNT162b2 vaccine-elicited neutralizing antibodies against authentic B.1, B.1.1.7, B.1.351, B.1.525 and P.1 SARS-CoV-2 variants. Emerg Microbes Infect 10:1241–1243. doi:10.1080/22221751.2021.1940305 34092181 PMC8216260

[16] Marshall JC, Murthy S, Diaz J, Adhikari NK, Angus DC, Arabi YM, Baillie K, Bauer M, Berry S, Blackwood B, et al.. 2020. A minimal common outcome measure set for COVID-19 clinical research. Lancet Infect Dis 20:e192–e197. doi:10.1016/S1473-3099(20)30483-7 32539990 PMC7292605

[17] Smith M, Abdesselem HB, Mullins M, Tan T-M, Nel AJM, Al-Nesf MAY, Bensmail I, Majbour NK, Vaikath NN, Naik A, et al.. 2021. Age, disease severity and ethnicity influence humoral responses in a multi-ethnic covid-19 cohort. Viruses 13:786. doi:10.3390/v13050786 33925055 PMC8146997

[18] Seow J, Graham C, Merrick B, Acors S, Pickering S, Steel KJA, Hemmings O, O’Byrne A, Kouphou N, Galao RP, et al.. 2020. Longitudinal observation and decline of neutralizing antibody responses in the three months following SARS-CoV-2 infection in humans. Nat Microbiol 5:1598–1607. doi:10.1038/s41564-020-00813-8 33106674 PMC7610833

[19] Luo YR, Chakraborty I, Yun C, Wu AHB, Lynch KL. 2021. Kinetics of severe acute respiratory syndrome coronavirus 2 (SARS-CoV-2) antibody avidity maturation and association with disease severity. Clin Infect Dis 73:e3095–e3097. doi:10.1093/cid/ciaa1389 32927483 PMC7543300

[20] Borremans B, Gamble A, Prager KC, Helman SK, McClain AM, Cox C, Savage V, Lloyd-Smith JO. 2020. Quantifying antibody kinetics and RNA detection during early-phase SARS-CoV-2 infection by time since symptom onset. Elife 9:1–27. doi:10.7554/eLife.60122 PMC7508557 32894217

[21] Rijkers G, Murk J-L, Wintermans B, van Looy B, van den Berge M, Veenemans J, Stohr J, Reusken C, van der Pol P, Reimerink J. 2020. Differences in antibody kinetics and functionality between severe and mild severe acute respiratory syndrome coronavirus 2 infections. J Infect Dis 222:1265–1269. doi:10.1093/infdis/jiaa463 32726417 PMC7454692

[22] Chen Y, Tong X, Li Y, Gu B, Yan J, Liu Y, Shen H, Huang R, Wu C. 2020. A comprehensive, longitudinal analysis of humoral responses specific to four recombinant antigens of SARS-CoV-2 in severe and non-severe COVID-19 patients. PLoS Pathog 16:e1008796. doi:10.1371/journal.ppat.1008796 32913364 PMC7482996

[23] Long Q-X, Liu B-Z, Deng H-J, Wu G-C, Deng K, Chen Y-K, Liao P, Qiu J-F, Lin Y, Cai X-F, et al.. 2020. Antibody responses to SARS-CoV-2 in patients with COVID-19. Nat Med 26:845–848. doi:10.1038/s41591-020-0897-1 32350462

[24] Seow J, Graham C, Merrick B, Acors S, Pickering S, Steel KJA, Steel O, O’Bryne A, Kouphou N, Pedro Galao R, et al.. 2020. Longitudinal evaluation and decline of antibody responses in SARS-CoV-2 infection. Nat Microbiol 5:1598–1607. doi:10.1038/s41564-020-00813-8 33106674 PMC7610833

[25] Prévost J, Gasser R, Beaudoin-Bussières G, Richard J, Duerr R, Laumaea A, Anand SP, Goyette G, Benlarbi M, Ding S, et al.. 2020. Cross-sectional evaluation of humoral responses against SARS-CoV-2 spike. Cell Rep Med 1:100126. doi:10.1016/j.xcrm.2020.100126 33015650 PMC7524645

[26] Long Q-X, Tang X-J, Shi Q-L, Li Q, Deng H-J, Yuan J, Hu J-L, Xu W, Zhang Y, Lv F-J, Su K, Zhang F, Gong J, Wu B, Liu X-M, Li J-J, Qiu J-F, Chen J, Huang A-L. 2020. Clinical and immunological assessment of asymptomatic SARS-CoV-2 infections. Nat Med 26:1200–1204. doi:10.1038/s41591-020-0965-6 32555424

[27] Isho B, Abe KT, Zuo M, Jamal AJ, Rathod B, Wang JH, Li Z, Chao G, Rojas OL, Bang YM, et al.. 2020. Persistence of serum and saliva antibody responses to SARS-CoV-2 spike antigens in COVID-19 patients. Sci Immunol 5:eabe5511. doi:10.1126/sciimmunol.abe5511 33033173 PMC8050884

[28] Robbiani DF, Gaebler C, Muecksch F, Lorenzi JCC, Wang Z, Cho A, Agudelo M, Barnes CO, Gazumyan A, Finkin S, et al.. 2020. Convergent antibody responses to SARS-CoV-2 in convalescent individuals. Nature 584:437–442. doi:10.1038/s41586-020-2456-9 32555388 PMC7442695

[29] Klein SL, Pekosz A, Park H-S, Ursin RL, Shapiro JR, Benner SE, Littlefield K, Kumar S, Naik HM, Betenbaugh MJ, Shrestha R, Wu AA, Hughes RM, Burgess I, Caturegli P, Laeyendecker O, Quinn TC, Sullivan D, Shoham S, Redd AD, Bloch EM, Casadevall A, Tobian AAR. 2020. Sex, age, and hospitalization drive antibody responses in a COVID-19 convalescent plasma donor population. J Clin Invest 130:6141–6150. doi:10.1172/JCI142004 32764200 PMC7598041

[30] Iyer AS, Jones FK, Nodoushani A, Kelly M, Becker M, Slater D, Mills R, Teng E, Kamruzzaman M, Garcia-Beltran WF, et al.. 2020. Persistence and decay of human antibody responses to the receptor binding domain of SARS-CoV-2 spike protein in COVID-19 patients. Sci Immunol 5:eabe0367. doi:10.1126/sciimmunol.abe0367 33033172 PMC7857394

[31] Legros V, Denolly S, Vogrig M, Boson B, Siret E, Rigaill J, Pillet S, Grattard F, Gonzalo S, Verhoeven P, Allatif O, Berthelot P, Pélissier C, Thiery G, Botelho-Nevers E, Millet G, Morel J, Paul S, Walzer T, Cosset F-L, Bourlet T, Pozzetto B. 2021. A longitudinal study of SARS-CoV-2-infected patients reveals a high correlation between neutralizing antibodies and COVID-19 severity. Cell Mol Immunol 18:318–327. doi:10.1038/s41423-020-00588-2 33408342 PMC7786875

[32] Guthmiller JJ, Stovicek O, Wang J, Changrob S, Li L, Halfmann P, Zheng N-Y, Utset H, Stamper CT, Dugan HL, Miller WD, Huang M, Dai Y-N, Nelson CA, Hall PD, Jansen M, Shanmugarajah K, Donington JS, Krammer F, Fremont DH, Joachimiak A, Kawaoka Y, Tesic V, Madariaga ML, Wilson PC. 2021. SARS-CoV-2 infection severity is linked to superior humoral immunity against the spike. mBio 12:1–13. doi:10.1128/mBio.02940-20 PMC7845638 33468695

[33] Garcia-Beltran WF, Lam EC, Astudillo MG, Yang D, Miller TE, Feldman J, Hauser BM, Caradonna TM, Clayton KL, Nitido AD, Murali MR, Alter G, Charles RC, Dighe A, Branda JA, Lennerz JK, Lingwood D, Schmidt AG, Iafrate AJ, Balazs AB. 2021. COVID-19-neutralizing antibodies predict disease severity and survival. Cell 184:476–488. doi:10.1016/j.cell.2020.12.015 33412089 PMC7837114

[34] Wang Y, Zhang L, Sang L, Ye F, Ruan S, Zhong B, Song T, Alshukairi AN, Chen R, Zhang Z, et al.. 2020. Kinetics of viral load and antibody response in relation to COVID-19 severity. J Clin Invest 130:5235–5244. doi:10.1172/JCI138759 32634129 PMC7524490

[35] Imai K, Kitagawa Y, Tabata S, Kubota K, Nagura-Ikeda M, Matsuoka M, Miyoshi K, Sakai J, Ishibashi N, Tarumoto N, Takeuchi S, Ito T, Maesaki S, Tamura K, Maeda T. 2021. Antibody response patterns in COVID-19 patients with different levels of disease severity in Japan. J Med Virol 93:3211–3218. doi:10.1002/jmv.26899 33620098 PMC8014305

[36] Shrock E, Fujimura E, Kula T, Timms RT, Lee I-H, Leng Y, Robinson ML, Sie BM, Li MZ, Chen Y, et al.. 2020. Viral epitope profiling of COVID-19 patients reveals cross-reactivity and correlates of severity. Science 370:eabd4250. doi:10.1126/science.abd4250 32994364 PMC7857405

[37] Russell CD, Lone NI, Baillie JK. 2023. Comorbidities, multimorbidity and COVID-19. Nat Med 29:334–343. doi:10.1038/s41591-022-02156-9 36797482

[38] Yu KKQ, Fischinger S, Smith MT, Atyeo C, Cizmeci D, Wolf CR, Layton ED, Logue JK, Aguilar MS, Shuey K, Loos C, Yu J, Franko N, Choi RY, Wald A, Barouch DH, Koelle DM, Lauffenburger D, Chu HY, Alter G, Seshadri C. 2021. Comorbid illnesses are associated with altered adaptive immune responses to SARS-CoV-2. JCI Insight 6:e146242. doi:10.1172/jci.insight.146242 33621211 PMC8026190

[39] Ko J-H, Müller MA, Seok H, Park GE, Lee JY, Cho SY, Ha YE, Baek JY, Kim SH, Kang J-M, Kim Y-J, Jo IJ, Chung CR, Hahn M-J, Drosten C, Kang C-I, Chung DR, Song J-H, Kang E-S, Peck KR. 2017. Serologic responses of 42 MERS-coronavirus-infected patients according to the disease severity. Diagn Microbiol Infect Dis 89:106–111. doi:10.1016/j.diagmicrobio.2017.07.006 28821364 PMC7127792

[40] Ibarrondo FJ, Fulcher JA, Goodman-Meza D, Elliott J, Hofmann C, Hausner MA, Ferbas KG, Tobin NH, Aldrovandi GM, Yang OO. 2020. Rapid decay of anti–SARS-CoV-2 antibodies in persons with mild Covid-19. N Engl J Med 383:1085–1087. doi:10.1056/NEJMc2025179 32706954 PMC7397184

[41] Cohen KW, Linderman SL, Moodie Z, Czartoski J, Lai L, Mantus G, Norwood C, Nyhoff LE, Edara VV, Floyd K, et al.. 2021. Longitudinal analysis shows durable and broad immune memory after SARS-CoV-2 infection with persisting antibody responses and memory B and T cells. medRxiv. doi:10.1101/2021.04.19.21255739 PMC8253687 34250512

[42] Zhang Z, Mateus J, Coelho CH, Dan JM, Moderbacher CR, Gálvez RI, Cortes FH, Grifoni A, Tarke A, Chang J, Escarrega EA, Kim C, Goodwin B, Bloom NI, Frazier A, Weiskopf D, Sette A, Crotty S. 2022. Humoral and cellular immune memory to four COVID-19 vaccines. Cell 185:2434–2451. doi:10.1016/j.cell.2022.05.022 35764089 PMC9135677

[43] Acevedo ML, Gaete-Argel A, Alonso-Palomares L, de Oca MM, Bustamante A, Gaggero A, Paredes F, Cortes CP, Pantano S, Martínez-Valdebenito C, Angulo J, Le Corre N, Ferrés M, Navarrete MA, Valiente-Echeverría F, Soto-Rifo R. 2022. Differential neutralizing antibody responses elicited by CoronaVac and BNT162b2 against SARS-CoV-2 Lambda in Chile. Nat Microbiol 7:524–529. doi:10.1038/s41564-022-01092-1 35365787

[44] Goel RR, Apostolidis SA, Painter MM, Mathew D, Pattekar A, Kuthuru O, Gouma S, Hicks P, Meng W, Rosenfeld AM, et al.. 2021. Distinct antibody and memory B cell responses in SARS-CoV-2 naïve and recovered individuals after mRNA vaccination. Sci Immunol 6:1–19. doi:10.1126/sciimmunol.abi6950 PMC8158969 33858945

[45] Stamatatos L, Czartoski J, Wan Y-H, Homad LJ, Rubin V, Glantz H, Neradilek M, Seydoux E, Jennewein MF, MacCamy AJ, Feng J, Mize G, De Rosa SC, Finzi A, Lemos MP, Cohen KW, Moodie Z, McElrath MJ, McGuire AT. 2021. mRNA vaccination boosts cross-variant neutralizing antibodies elicited by SARS-CoV-2 infection. Science 372:1413–1418. doi:10.1126/science.abg9175 33766944 PMC8139425

[46] Tada T, Zhou H, Dcosta BM, Samanovic MI, Chivukula V, Herati RS, Hubbard SR, Mulligan MJ, Landau NR. 2022. Increased resistance of SARS-CoV-2 Omicron variant to neutralization by vaccine-elicited and therapeutic antibodies. EBioMedicine 78:103944. doi:10.1016/j.ebiom.2022.103944 35465948 PMC9021600

[47] Evans JP, Zeng C, Carlin C, Lozanski G, Saif LJ, Oltz EM, Gumina RJ, Liu S-L. 2022. Neutralizing antibody responses elicited by SARS-CoV-2 mRNA vaccination wane over time and are boosted by breakthrough infection. Sci Transl Med 14:eabn8057. doi:10.1126/scitranslmed.abn8057 35166573 PMC8939766

[48] Pooley N, Abdool Karim SS, Combadière B, Ooi EE, Harris RC, El Guerche Seblain C, Kisomi M, Shaikh N. 2023. Durability of vaccine-induced and natural immunity against COVID-19: a narrative review. Infect Dis Ther 12:367–387. doi:10.1007/s40121-022-00753-2 36622633 PMC9828372

[49] Chiarella SE, Jenkins SM, Smith CY, Prasad V, Shakuntulla F, Ahluwalia V, Iyer VN, Theel ES, Joshi AY. 2022. Predictors of seroconversion after coronavirus disease 2019 vaccination. Ann Allergy Asthma Immunol 129:189–193. doi:10.1016/j.anai.2022.05.026 35640775 PMC9144839

[50] Seery V, Raiden S, Russo C, Borda M, Herrera L, Uranga M, Varese A, Marcó Del Pont M, Chirino C, Erramuspe C, et al.. 2022. Antibody response against SARS-CoV-2 variants of concern in children infected with pre-Omicron variants: an observational cohort study. EBioMedicine 83:104230. doi:10.1016/j.ebiom.2022.104230 35988465 PMC9387350

[51] Muena NA, García-Salum T, Pardo-Roa C, Avendaño MJ, Serrano EF, Levican J, Almonacid LI, Valenzuela G, Poblete E, Strohmeier S, Salinas E, Muñoz A, Haslwanter D, Dieterle ME, Jangra RK, Chandran K, González C, Riquelme A, Krammer F, Tischler ND, Medina RA. 2022. Induction of SARS-CoV-2 neutralizing antibodies by CoronaVac and BNT162b2 vaccines in naïve and previously infected individuals. EBioMedicine 78:103972. doi:10.1016/j.ebiom.2022.103972 35366624 PMC8965458

[52] Abu Jabal K, Ben-Amram H, Beiruti K, Batheesh Y, Sussan C, Zarka S, Edelstein M. 2021. Impact of age, ethnicity, sex and prior infection status on immunogenicity following a single dose of the BNT162b2 MRNA COVID-19 vaccine: real-world evidence from healthcare workers, Israel, December 2020 to January 2021. Euro Surveill 26:2100096. doi:10.2807/1560-7917.ES.2021.26.6.2100096 33573712 PMC7879501

[53] Levi R, Azzolini E, Pozzi C, Ubaldi L, Lagioia M, Mantovani A, Rescigno M. 2021. One dose of SARS-CoV-2 vaccine exponentially increases antibodies in individuals who have recovered from symptomatic COVID-19. J Clin Invest 131:e149154. doi:10.1172/JCI149154 33956667 PMC8203458

[54] Samanovic MI, Cornelius AR, Gray-Gaillard SL, Allen JR, Karmacharya T, Wilson JP, Hyman SW, Tuen M, Koralov SB, Mulligan MJ, Herati RS. 2022. Robust immune responses are observed after one dose of BNT162b2 mRNA vaccine dose in SARS-CoV-2-experienced individuals. Sci Transl Med 14:eabi8961. doi:10.1126/scitranslmed.abi8961 34874183 PMC9248013

[55] Saadat S, Rikhtegaran Tehrani Z, Logue J, Newman M, Frieman MB, Harris AD, Sajadi MM. 2021. Binding and neutralization antibody titers after a single vaccine dose in health care workers previously infected with SARS-CoV-2. JAMA 325:1467–1469. doi:10.1001/jama.2021.3341 33646292 PMC7922233

[56] Krammer F, Srivastava K, Simon V, the PARIS team. 2021. Robust spike antibody responses and increased reactogenicity in seropositive individuals after a single dose of SARS-CoV-2 mRNA vaccine. medRxiv. doi:10.1101/2021.01.29.21250653

[57] Singanayagam A, Hakki S, Dunning J, Madon KJ, Crone MA, Koycheva A, Derqui-Fernandez N, Barnett JL, Whitfield MG, Varro R, Charlett A, Kundu R, Fenn J, Cutajar J, Quinn V, Conibear E, Barclay W, Freemont PS, Taylor GP, Ahmad S, Zambon M, Ferguson NM, Lalvani A, ATACCC Study Investigators. 2022. Community transmission and viral load kinetics of the SARS-CoV-2 delta (B.1.617.2) variant in vaccinated and unvaccinated individuals in the UK: a prospective, longitudinal, cohort study. Lancet Infect Dis 22:183–195. doi:10.1016/S1473-3099(21)00648-4 34756186 PMC8554486

[58] Kissler SM, Fauver JR, Mack C, Tai CG, Breban MI, Watkins AE, Samant RM, Anderson DJ, Metti J, Khullar G, Baits R, MacKay M, Salgado D, Baker T, Dudley JT, Mason CE, Ho DD, Grubaugh ND, Grad YH. 2021. Viral dynamics of SARS-CoV-2 variants in vaccinated and unvaccinated persons. N Engl J Med 385:2489–2491. doi:10.1056/NEJMc2102507 34941024 PMC8693673

[59] Tan ST, Kwan AT, Rodríguez-Barraquer I, Singer BJ, Park HJ, Lewnard JA, Sears D, Lo NC. 2023. Infectiousness of SARS-CoV-2 breakthrough infections and reinfections during the Omicron wave. Nat Med 29:358–365. doi:10.1038/s41591-022-02138-x 36593393 PMC9974584

[60] Planas D, Veyer D, Baidaliuk A, Staropoli I, Guivel-Benhassine F, Rajah MM, Planchais C, Porrot F, Robillard N, Puech J, et al.. 2021. Reduced sensitivity of SARS-CoV-2 variant Delta to antibody neutralization. Nature 596:276–280. doi:10.1038/s41586-021-03777-9 34237773

[61] Anti-SARS-CoV-2 ELISA (IgG) - Instructions for use. Accessed 6 April 2024. https://cdnmedia.eurofins.com/eurofins-us/media/1711222/ei_2606g_a_us_c01_igg_ce.pdf.

[62] Gededzha MP, Mampeule N, Jugwanth S, Zwane N, David A, Burgers WA, Blackburn JM, Grove JS, George JA, Sanne I, Scott L, Stevens W, Mayne ES. 2021. Performance of the EUROIMMUN Anti-SARS-CoV-2 ELISA Assay for detection of IgA and IgG antibodies in South Africa. PLoS One 16:e0252317. doi:10.1371/journal.pone.0252317 34161348 PMC8221517

[63] Beavis KG, Matushek SM, Abeleda APF, Bethel C, Hunt C, Gillen S, Moran A, Tesic V. 2020. Evaluation of the EUROIMMUN anti-SARS-CoV-2 ELISA assay for detection of IgA and IgG antibodies. J Clin Virol 129:104468. doi:10.1016/j.jcv.2020.104468 32485620 PMC7255182

[64] Burn Aschner C, Muthuraman K, Kucharska I, Cui H, Prieto K, Nair MS, Wang M, Huang Y, Christie-Holmes N, Poon B, Lam J, Sultana A, Kozak R, Mubareka S, Rubinstein JL, Rujas E, Treanor B, Ho DD, Jetha A, Julien J-P. 2023. A multi-specific, multi-affinity antibody platform neutralizes sarbecoviruses and confers protection against SARS-CoV-2 in vivo. Sci Transl Med 15:eadf4549. doi:10.1126/scitranslmed.adf4549 37224226

[65] Corman VM, Landt O, Kaiser M, Molenkamp R, Meijer A, Chu DK, Bleicker T, Brünink S, Schneider J, Schmidt ML, Mulders DG, Haagmans BL, van der Veer B, van den Brink S, Wijsman L, Goderski G, Romette J-L, Ellis J, Zambon M, Peiris M, Goossens H, Reusken C, Koopmans MP, Drosten C. 2020. Detection of 2019 novel coronavirus (2019-nCoV) by real-time RT-PCR. Euro Surveill 25:2000045. doi:10.2807/1560-7917.ES.2020.25.3.2000045 31992387 PMC6988269

[66] Rujas E, Kucharska I, Tan YZ, Benlekbir S, Cui H, Zhao T, Wasney GA, Budylowski P, Guvenc F, Newton JC, et al.. 2021. Multivalency transforms SARS-CoV-2 antibodies into ultrapotent neutralizers. Nat Commun 12:3661. doi:10.1038/s41467-021-23825-2 34135340 PMC8209050

[67] Crawford KHD, Eguia R, Dingens AS, Loes AN, Malone KD, Wolf CR, Chu HY, Tortorici MA, Veesler D, Murphy M, Pettie D, King NP, Balazs AB, Bloom JD. 2020. Protocol and reagents for pseudotyping lentiviral particles with SARS-CoV-2 spike protein for neutralization assays. Viruses 12:513. doi:10.3390/v12050513 32384820 PMC7291041

