# Supplementary material for: Persistence and decay of neutralizing antibody responses elicited by SARS-CoV-2 infection and hybrid immunity in a Canadian cohort
Source: Microbiol Spectr. 2025 Feb 19;13(4):e01333-24. doi: 10.1128/spectrum.01333-24 (PMC11960127; doi:10.1128/spectrum.01333-24)
Supplement: Figure S1 and S2 — Antibody responses from naturally infected patients wane over time and the correlation between anti-S1 IgGS and neutralizing antibodies. [file spectrum.01333-24-s0001.docx]

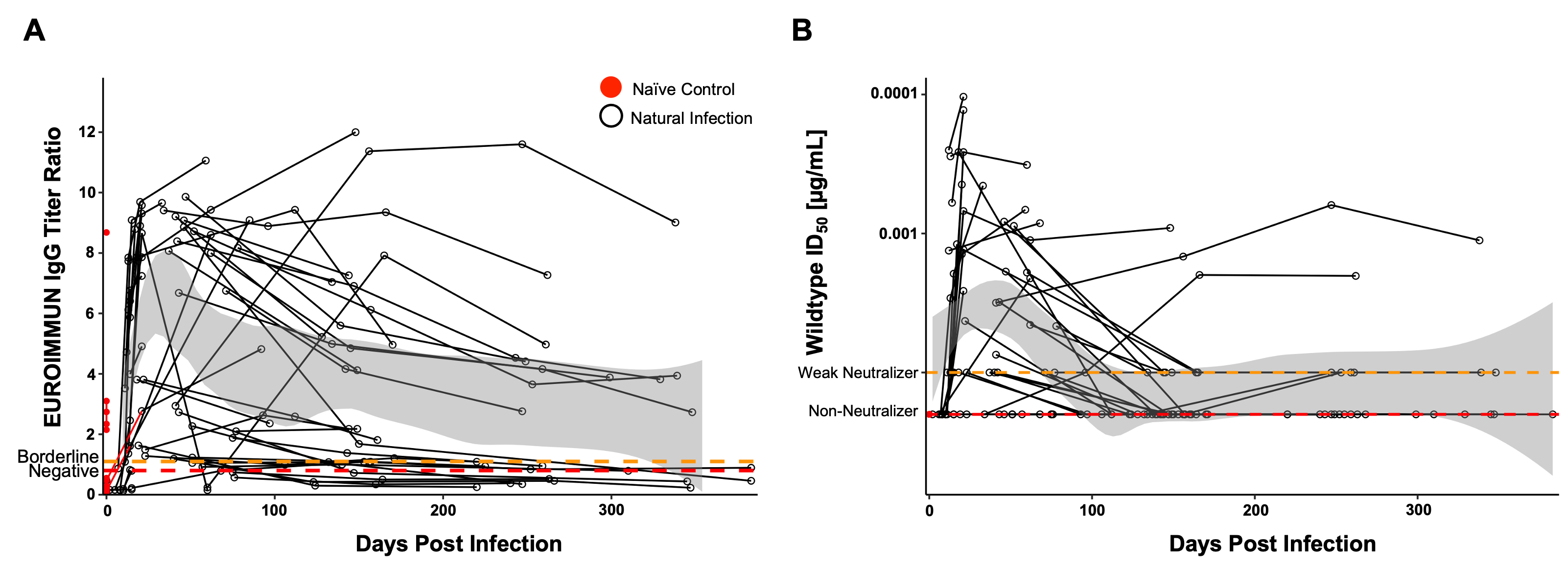


**Supplemental Figure 1: Antibody responses from naturally infected patients wanes over time.**

Analysis of the changes in the IgG-spike levels (**A**) and PsV neutralization titer (ID_50_) (**B**) in patients profiled more than twice, in comparison to the relative levels in pre-COVID negative controls represented by red dots. **A**) Black dots represent individual serum samples collected at the indicated times, and the samples from the same patients are connected by the lines. A non-parametric loess function is shown where a span of 0.5 is applied, with the grey shade representing the 95% confidence interval. The red dotted line at a ratio of 0.8 signifies those points below this line is negative for anti-Spike IgGs. The orange dotted line at a ratio of 1.1 signifies that points between the orange and red dotted line are borderline for anti-Spike IgGs. **B**) Each black point represents a measurement of 50% neutralizing titer (ID_50_). A non-parametric loess function is shown where a span of 0.6 is applied, with the grey shade representing the 95% confidence interval. The orange dotted line at 0.01 μg/mL represents weak neutralizing samples that can neutralize the WT PsV at a dilution of 1:50 but we are unable to fit a proper sigmoidal curve to the data set, the red dotted line at an ID_50_ value of 0.02 μg/mL represents non-neutralizing samples, which are samples that do not meet any of the criteria to assign an ID_50_ value to the sample.


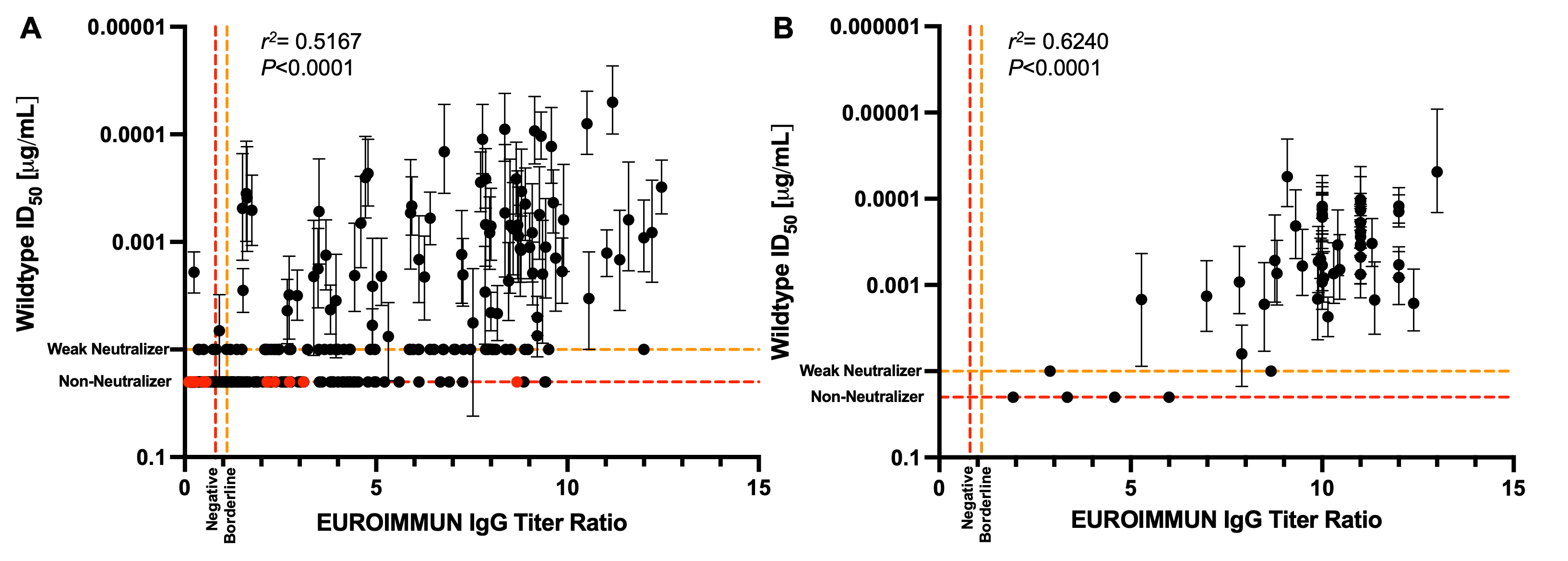


**Supplemental Figure 2: Correlation between titres of spike-S1 IgG and neutralizing antibodies.**

Log-linear regression analyses were performed on Wildtype (D614G) PsV neutralization titers versus anti-S1 IgGs titers in the cohort before vaccination which includes the naïve controls and natural infection only cohort A) (**A**) and after vaccination which includes the naturally infected and vaccinated (hybrid immunity - cohort B) (**B**). The red dotted line at a ratio of 0.8 on the x-axis signifies those points below this line is negative for anti-Spike IgGs. The orange dotted line at a ratio of 1.1 on the x-axis signifies that points between the orange and red dotted line are borderline for anti-Spike IgGs. The orange dotted line at 0.01 μg/mL on the y-axis represents weak neutralizing samples that can neutralize the WT PsV at a dilution of 1:50 but we are unable to fit a proper sigmoidal curve to the data set, the red dotted line at an ID_50_ value of 0.02 μg/mL on the y-axis represents non-neutralizing samples, which are samples that do not meet any of the criteria to assign an ID_50_ value to the sample. Pearson correlations were calculated and *R^2^* and *p* values are indicated.
